# Admixture between Ancient Lineages, Selection, and the Formation of Sympatric Stickleback Species-Pairs

**DOI:** 10.1093/molbev/msz161

**Published:** 2019-07-16

**Authors:** Laura L Dean, Isabel S Magalhaes, Andrew Foote, Daniele D’Agostino, Suzanne McGowan, Andrew D C MacColl

**Affiliations:** 1 School of Life Sciences, The University of Nottingham, Nottingham, United Kingdom; 2 Department of Life Sciences, Whitelands College, University of Roehampton, London, United Kingdom; 3 Molecular Ecology and Fisheries Genetics Laboratory, Bangor University, Bangor, United Kingdom; 4 School of Geography, The University of Nottingham, Nottingham, United Kingdom

**Keywords:** *Gasterosteus aculeatus*, reproductive isolation, three-spined stickleback, admixture, adaptive radiation, speciation

## Abstract

Ecological speciation has become a popular model for the development and maintenance of reproductive isolation in closely related sympatric pairs of species or ecotypes. An implicit assumption has been that such pairs originate (possibly with gene flow) from a recent, genetically homogeneous ancestor. However, recent genomic data have revealed that currently sympatric taxa are often a result of secondary contact between ancestrally allopatric lineages. This has sparked an interest in the importance of initial hybridization upon secondary contact, with genomic reanalysis of classic examples of ecological speciation often implicating admixture in speciation. We describe a novel occurrence of unusually well-developed reproductive isolation in a model system for ecological speciation: the three-spined stickleback (*Gasterosteus aculeatus*), breeding sympatrically in multiple lagoons on the Scottish island of North Uist. Using morphological data, targeted genotyping, and genome-wide single-nucleotide polymorphism data, we show that lagoon resident and anadromous ecotypes are strongly reproductively isolated with an estimated hybridization rate of only ∼1%. We use palaeoecological and genetic data to test three hypotheses to explain the existence of these species-pairs. Our results suggest that recent, purely ecological speciation from a genetically homogeneous ancestor is probably not solely responsible for the evolution of species-pairs. Instead, we reveal a complex colonization history with multiple ancestral lineages contributing to the genetic composition of species-pairs, alongside strong disruptive selection. Our results imply a role for admixture upon secondary contact and are consistent with the recent suggestion that the genomic underpinning of ecological speciation often has an older, allopatric origin.

## Introduction

The sympatric coexistence of closely related but reproductively isolated “species-pairs” represents a biological conundrum because it is difficult to explain how divergence is maintained in the absence of obvious barriers to gene flow. Ecological speciation, in which ecologically dependent natural selection drives the evolution of variation in adaptive traits that also influence reproductive isolation between ecotypes, has become a popular model to account for this problem (Rundle and Nosil 2005; Schluter 1996, 2009; Nosil 2012). However, it is becoming increasingly apparent that ecologically derived selection rarely results in complete speciation, but rather tends to cause only partial divergence, either with reduced (but still some) genome-wide gene flow between, compared with within ecotypes, or only certain regions of the genome being unaffected by gene flow (Hendry 2009; Hendry et al. 2009).

Increasing resolution of genomic data have made it possible to untangle how the demographic history of species-pairs varies across the genome, bringing potential for a much clearer picture of how historic periods of secondary contact and admixture events have shaped the evolutionary trajectory of species (Sousa and Hey 2013). Genomic data are beginning to suggest that stronger recent divergence is often underlain by more ancient genetic incompatibilities, or adaptive variation, that evolved in allopatry (Seehausen et al. 2014; Marques et al. 2019). As such, many currently sympatric taxa, which appear to have diverged in situ, have later been found to be the result of secondary contact following a long period of allopatry (Bernatchez and Dodson 1990; Feder et al. 2003; Kuehne et al. 2007; Foote and Morin 2015; Lucek et al. 2018). These ancient alleles contributing to speciation can be a result of standing genetic variation that is reused to seed new ecological divergences and reproductive barriers (Feder et al. 2005; Jones, Grabherr, et al. 2012) or admixture/introgression that provides genetic material for both adaptation and reproductive isolation in the face of gene flow (Seehausen 2004; Keller et al. 2013; Marques et al. 2019). The importance of genetic admixture upon secondary contact for speciation has long been known in plants (Grant 1971; Rieseberg et al. 2003; Soltis and Soltis 2009), but in the light of the realization that purely ecological speciation may be rare (Hendry 2009; Wang et al. 2013), there has been a recent, rising awareness that hybridization and admixture events are often also involved in animal speciation (Mallet 2007; Abbott et al. 2013; Feder et al. 2013). Many classic examples of ecological speciation, such as Darwin’s finches (Grant and Grant 2009) and adaptive radiations such as the cichlids of Lake Victoria (Kocher 2004; Terai et al. 2006) and the *Anopheles* species-complex (Simard et al. 2009), are turning out either to be cryptic cases of homoploid hybrid speciation (Lamichhaney et al. 2018) or to have involved genetic admixture events (Fontaine et al. 2015; Meier et al. 2017, 2018; Marques et al. 2019). In this new genomic era, it is therefore necessary to reassess the mechanisms responsible for speciation, particularly in model systems of adaptive radiation.

Divergence among populations of three-spined stickleback (*Gasterosteus aculeatus* L., hereafter “stickleback”) is common throughout their Holarctic range (Bell et al. 2004). Truly marine, sea-spawning stickleback have repeatedly colonized coastal brackish and freshwater habitats following the Pleistocene glacial retreat, giving rise to anadromous (migratory fish which spend most of their lives in the sea but migrate into fresh or brackish water to spawn), lagoon resident (fish that live year-round in shallow brackish coastal lagoons), and freshwater resident (fish that live year round in freshwater lakes and streams) ecotypes (Taylor and McPhail 2000; McKinnon et al. 2004). Very little is known about truly marine stickleback (Ahnelt 2018), but studies of anadromous and resident (resident in either fresh or brackish water) populations show that differences in ecological selection pressures acting between oceanic and enclosed waters (e.g., lakes, streams, and lagoons) have shaped the replicated heritable changes in morphology and behavior that are associated with these parallel transitions (Schluter and McPhail 1992; McKinnon and Rundle 2002; McKinnon et al. 2004; Schluter et al. 2004; Jones, Grabherr, et al. 2012). While there is substantial evidence of divergent natural selection and genetic differentiation between anadromous and resident ecotypes (Hagen 1967; McKinnon and Rundle 2002; Von Hippel and Weigner 2004; Jones et al. 2006), extensive hybridization is apparent across most parapatric contact zones (Heuts 1947; Hagen 1967; Hay and McPhail 2000; Jones et al. 2006). There are only a handful of cases in which admixed, morphologically intermediate individuals are completely absent (Ziuganov 1995; Karve et al. 2008) and fewer still with direct genetic evidence for the absence of admixture (Drevecky et al. 2013). Therefore, as with many other species, it has been concluded that ecologically mediated selection alone is not sufficient in this system for speciation to reach completion in the face of gene flow (Hendry 2009; Schluter and Conte 2009).

There are a few rare cases in which speciation has progressed further along the continuum of reproductive isolation in stickleback. The most unambiguous example of speciation to completion is between two marine forms: Pacific Ocean and Japan Sea stickleback (Higuchi and Goto 1996). This is a clear example of intrinsic genetic speciation that is allopatric and ancient in origin (Kitano et al. 2007, 2009) and has occurred in the face of ongoing gene flow (Ravinet et al. 2018). A second example is freshwater benthic-limnetic species-pairs in multiple lakes around the Strait of Georgia, BC, Canada, which show strong reproductive isolation with unusually low (∼5%) hybridization (McPhail 1992; Gow et al. 2006, 2008). The origins of benthic–limentic pairs are much less clear. Ecologically mediated selection is important for maintaining distinct benthic and limnetic ecotypes (Schluter and McPhail 1992; Schluter 1996; McKinnon and Rundle 2002), but is unlikely to be wholly accountable for speciation (Hendry 2009). It was initially thought that benthic–limnetic pairs evolved independently in each lake from a single, homogeneous “stock” marine population, and the effects of ecological divergence were exaggerated because postglacial fluctuations in relative sea-level (RSL) caused a “double-invasion” of marine fish, with an intermediate period of spatial isolation in which gene-flow was prevented (McPhail 1993; Taylor and McPhail 2000; Rundle and Schluter 2004). This is the “classic” model proposed to explain cases of unusually strong reproductive isolation in many postglacial fish (Ferguson and Taggart 1991; Schluter 1996; Volpe and Ferguson 1996; Nesbo et al. 1999), but it is rarely, if ever, empirically tested. Furthermore, RSL reconstructions for the Strait of Georgia are not consistent with a double-invasion for benthic–limnetic pairs (Friele and Hutchinson 1993; Josenhans et al. 1997; Hutchinson et al. 2004) and Jones, Chan, et al. (2012) showed that some of the adaptive genetic variation in each lake arises from shared adaptive variants, rather than from novel mutations that have arisen separately in each lake, suggesting a more important role for allopatric adaptive divergence and reuse of standing genetic variation in the evolution of benthic–limnetic species-pairs. Some prior genetic differentiation is therefore probably necessary for speciation to progress beyond low level reproductive isolation under conditions of ecologically derived selection in stickleback.

**Fig. 1.**
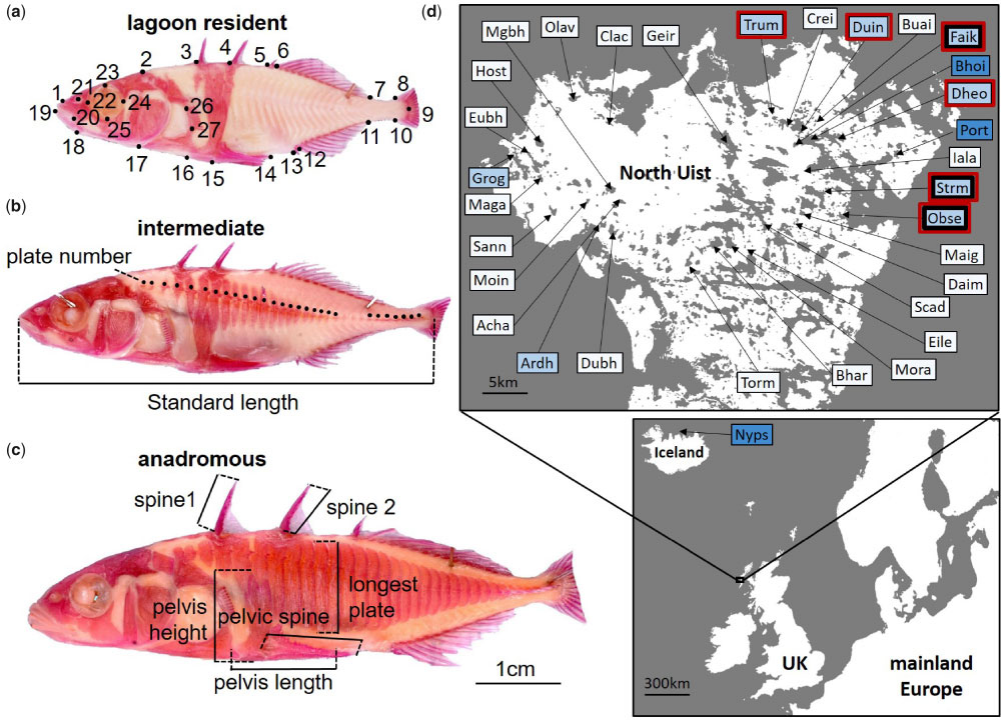
Examples of lagoon resident (*a*), intermediate (*b*), and anadromous (*c*) phenotypes alongside a map showing sampling locations (*d*). Images show stickleback that have been stained with Alizarin red to highlight external skeletal structures. (*a*) Shows the positions of 27 landmarks used in the geometric morphometric analysis of body shape, (*b*) shows how lateral plate counts and measurements of standard length were taken, and (*c*) how measurements of all continuous body armor variables were taken. (*d*) Shows the locations of all sample sites from which data were used, with marine (absolute conductivity >35,000 μS/cm) locations labeled in dark blue, brackish (absolute conductivity 20,000–35,000 μS/cm) locations labeled in midblue and freshwater (absolute conductivity <500 μS/cm) labeled light blue. A thick black border represents sites for which marine inundation history was reconstructed and a thick red border indicate sites containing species-pairs examined in this article (see supplementary table S1, Supplementary Material online, for a detailed description of sampling sites and which analyses each site was used for).

The island of North Uist (hereafter “Uist”), Scottish Western Isles, like most of the rest of northern Europe, was likely colonized by marine stickleback following the melting of ice sheets ∼16,000 YBP (Colosimo et al. 2005; Jones, Grabherr, et al. 2012). The island comprises a series of complex isolated or interconnected freshwater lakes and brackish coastal lagoons, which cover almost one-third of the land surface of the island, making it ideal for studying the oceanic-resident radiation of stickleback. Here, we use high resolution, genome-wide single-nucleotide polymorphism (SNP) data alongside targeted genotyping and morphological analysis to identify apparently stable, strongly isolated, sympatrically breeding anadromous–lagoon resident stickleback species-pairs in multiple brackish coastal lagoons on Uist (see fig. 1*a–c* for phenotypic examples of species-pair parental and intermediate phenotypes). We then combine genetic and palaeoecological data to test three possible hypotheses, which are not mutually exclusive, to explain the origin of these previously unexplored species-pairs: 1) the “classic” stickleback model, in which multiple colonizations from a single, homogeneous marine population occurred as a result of a double-invasion facilitated by changes in RSL, and speciation occurred purely as a result of recent ecological divergent selection that occurred in situ during the Holocene. This possibility is supported by some previous evidence that suggests a spike (“high-stand”) in RSL immediately after deglaciation in the Hebrides, followed by RSL receding until ∼10,000–14,000 YBP, before rising again to the present day (Jordan et al. 2010). However, it is also clear that local patterns of sea-level change can be very variable as a result of differences in glaciation and solid geology. This hypothesis makes four predictions. First, there is evidence for strong, ecologically based selection between divergent ecotypes; second, the species-pairs originated from multiple colonizations from a genetically homogeneous “stock” marine population, with no prior genetic or behavioral isolation (Rundle and Schluter 2004). Third, the species-pairs are postglacial in age as divergence can only have occurred since the deglaciation of Uist ∼16,000 YBP (Ballantyne 2010), and fourth, the lagoons in question experienced a double peak in RSL, leading to two periods of marine inundation of some lagoons, depending on altitude, during that time.

2) Alternatively, genetic divergence evolved in allopatry, prior to colonization, and Uist experienced a double-invasion that was brought about by differential arrival times of different lineages/ecotypes (and therefore not necessarily related to RSL change), leading to secondary contact between two already distinct lineages. There is evidence that the west coast of Scotland may be a rare contact zone for ancient mitochondrial stickleback lineages that persisted in glacial refugia on either side of the Atlantic during the Pleistocene (Makinen and Merila 2008), making this a distinct possibility. In this case, it is possible that reproductive isolation may already have been well developed upon secondary contact, and species-pairs are directly descendent from two ancient allopatric marine lineages. This hypothesis makes four clear predictions: that Uist was colonized by multiple prediverged lineages; there is a differential genetic origin of ecotypes for example, as in Bernatchez and Dodson (1990); this prior genetic divergence significantly predates the last glacial retreat and there is no evidence for recent admixture between ecotypes.

3) Finally, prior allopatric divergence may have existed, as in (2), but rather than reproductive isolation having reached completion prior to secondary contact, species pairs could have formed as a direct result of admixture between lineages upon secondary contact. This hypothesis predicts that Uist was colonized by multiple, prediverged stickleback populations; that there is evidence for recent admixture between those populations and one or both of the species-pair ecotypes is genetically admixed in relation to putative parental populations. To test these three hypotheses, we reconstruct the Holocene marine inundation history of a series of coastal lagoons on Uist and use targeted mitochondrial sequencing alongside genome-wide SNP data to investigate the demographic history and genetic relationships of species-pairs inhabiting those lagoons. By consideration of putative mechanisms for these three hypotheses, we shed light on the underlying mechanisms of speciation in postglacial fish.

## Results

### Geographical Evidence for a “Double Invasion”

Assessing the role of spatial isolation in speciation is notoriously problematic because inferring spatial distributions of historic populations is difficult over long time-scales (Kozak et al. 2008). However, estimates of past lake-sea connectivity can be reconstructed using sediment elemental composition (Ziegler et al. 2008; Chague-Goff et al. 2016) and diatom assemblages (Fritz et al. 1991) from sediment deposited on the lakebed. This allows periods of potential colonization and spatial isolation for coastal aquatic species to be reconstructed and precisely dated, and is a dramatically underutilized resource in speciation research. There are no records of RSL change over the Holocene directly for North Uist, and therefore, we tested our hypothesis that a double-invasion could have been caused by changes in RSL by reconstructing Holocene lagoon-sea connectivity for a series of three lagoons, of varying elevation, containing stickleback species-pairs (Obse, Faik, and Strm, which had an elevation of 1.63, 1.16, and 0.92 m above datum, respectively, see locations highlighted with a thick black border in fig. 1*d* for geographical locations and supplementary table S1, Supplementary Material online, for detailed sample site information).

**Fig. 2.**
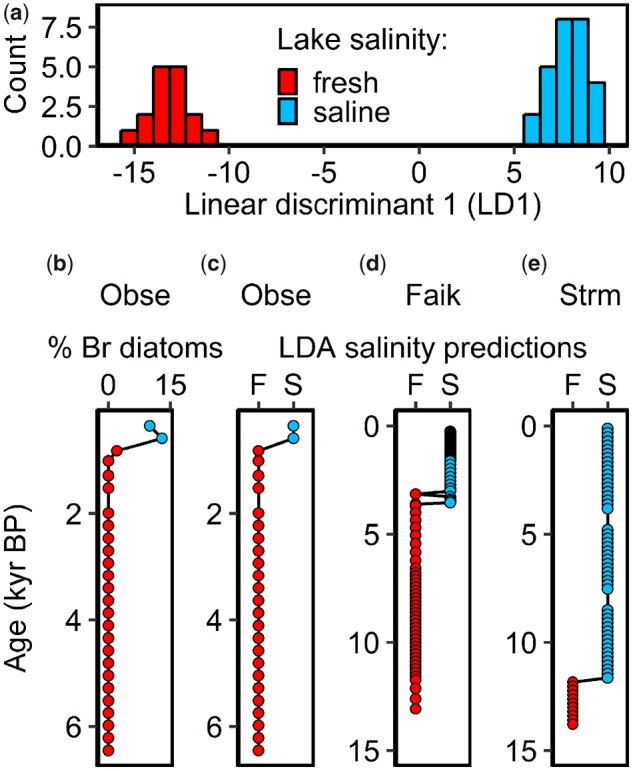
Salinity reconstructions for currently brackish North Uist lagoons. (a) Separation of freshwater (red bars, <250 μS/cm) and saline (blue bars, >20,000 μS/cm) waterbodies along linear discriminant one (LD1) of a linear discriminant analyses (LDA) based on sediment elemental composition used to classify long core sediment in (b-e). Models of past salinity for Obse (b-c), Faik (d), and Strm (e) (elevations: 1.63, 1.16, and 0.92m above datum, respectively) based on the percentage of brackish diatom species (% Br diatoms) and the predictions of a LDA of lake sediment elemental composition (LDA salinity predictions). For the LDA, red circles and “F” correspond to “freshwater” conditions and blue circles and “S” to “saline” conditions. For diatom count, blue circles indicate >5% brackish diatom species and red circles <5%. For salinity reconstructions in (b-e), age is estimated for Faik using a Bayesian age-depth model based on six radiocarbon dates (supplementary table S3, Supplementary Material online), implemented using the R package Bacon, and for all other cores by transposing the linear mean sedimentation rate in Faik (supplementary fig. S1, Supplementary Material online).

The elemental composition of surface sediments from five locations (Iala, Crei, Bhoi, Dheo, and Port), deposited under known salinity conditions, was used as a calibration series to create a discriminant function to predict the salinity under which older sediment was deposited. Surface (upper 20 cm) freshwater and marine (absolute water conductivity: <250 and >20,000 μS/cm, respectively) sediment deposits clustered distinctly and separately along a single linear discriminant axis (LD1, fig. 2*a*) and jack-knifed validation indicated that the linear discriminant analysis (LDA) was 100% accurate in classifying known sediment samples into the correct group (*n* = 43). The LDA predicted the salinity of all elemental samples from Obse, Faik, and Strm long cores with posterior probabilities of >0.99. LDA predictions indicated that all three currently brackish lagoons transitioned from freshwater conditions during the time spanned by our sediment sequences (fig. 2*b–e*), but with no previous evidence of a marine phase. Diatom species counts from Obse validated LDA predictions, indicating an identical freshwater to saline transition as predicted by the LDA, with freshwater diatom species throughout the majority of the core and brackish species appearing only in the top 20 cm of sediment (fig. 2*b*, for a full list of identified diatom species, see supplementary table S2, Supplementary Material online).

Radiocarbon dating of macrofossil material indicated that the Faik long core spanned the Holocene period with basal sediment ∼13,097–13,289 cal. YBP (supplementary table S3, Supplementary Material online). The sedimentation rate in Faik was 24.26 cm/ky and almost completely linear (supplementary fig. S1, Supplementary Material online). Estimates from transposing the sedimentation rate in Faik suggested that basal sediment in Strm was deposited ∼14,500 cal. YBP and in Obse 6,000–7,000 cal. YBP. Based on depth–age relationships, the transition to saline conditions in Obse probably occurred within the last 1,000 years with no indication of an older saline period (fig. 2*b* and *c*). The earliest indication of saline influx in Faik occurred between 3,294 and 3,364 cal. YBP and consistently saline conditions were reached by ∼2,793–3,244 cal. YBP (fig. 2*d* andsupplementary table S3, Supplementary Material online), again with no indication of a second, older saline period. Strm contained the deepest initial saline section, transitioning from freshwater ∼10,750 cal. YBP (fig. 2*e*) based on the same age–depth model and sedimentation rates as Faik. The stratified depth and estimated timing of these saline transitions across lagoons is consistent with elevation data from outlet sills, which revealed that Obse was the highest lagoon, followed by Faik, then Strm (1.63, 1.16, and 0.92 m above datum, respectively).

### Divergence in Species-Pairs

#### Morphological Differentiation

**Fig. 3.**
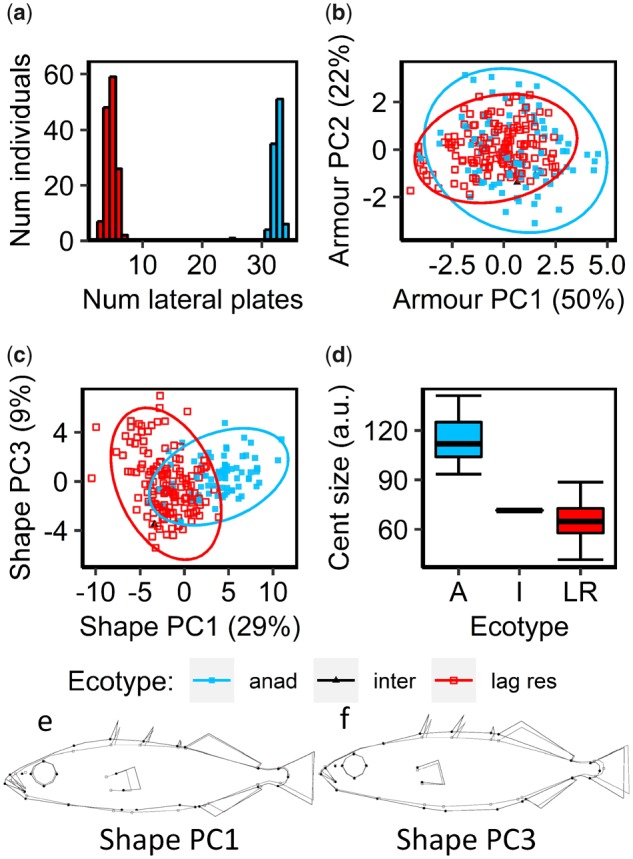
Morphological variation in North Uist stickleback species-pairs. (*a*) histogram showing lateral plate counts. (*b*) Distribution of phenotypes and their associated 95% confidence ellipses in a principal components analysis (PCA) of all size standardized, continuous body armor variables (see fig. 1*c* for continuous armor measurements). Principal component 1 (Armor PC1, explaining 50% of variation in the data) described an increase in the size of all armor variables and Armor PC2 (explaining 22% of variation in the data) described a relative increase in the size of the pelvis and decrease in spine length. (*c*) Distribution of phenotypes and their associated 95% confidence ellipses in a PCA of 56 body shape variables (derived from 27 landmarks, see fig. 1*a* for landmark positions). Shape PC1 (explaining 29% of variation in the data) described an increase in posterior body depth, mouth size, and a more rearward positioning of the pectoral fin. Shape PC3 (explaining 9% of variation in the data) largely described an increase in anterior body depth and shortening of the caudal peduncle. Shape changes for Shape PC1 and 3 are shown in warped outline drawings (*e*) and (*f*), respectively, with 1.5% scaling. (*d*) Box-plots showing centroid size, with error bars representing the standard error of the mean (SEM). (*a*)–(*f*) are based on analyses of 239 individuals from six lakes containing species-pairs (fig. 1*d* and supplementary table S1, Supplementary Material online).

Lagoon resident and anadromous stickleback differed consistently in body armor, shape, and size (fig. 3) across six species-pair lagoons (Faik, Obse, Duin, Trum Strm, and Dheo, see sites marked with red borders in fig. 1*d*). Morphological differences in species-pairs were largely consistent across sample sites (supplementary fig. S3, Supplementary Material online) and therefore results from all sites combined are discussed below. Firstly, anadromous ecotypes had considerably more lateral bony armor plates than lagoon residents (*t *=* *280.94, df = 230.65, *P *<* *0.0001, fig. 3*a*), with the two ecotypes possessing 31–34 plates and three to seven plates, respectively (hereafter referred to as “completely plated” and “low-plated” morphs). Initial sampling of 239 individuals, euthanized and stained for detailed morphological characterization, identified only one partially plated individual (defined, for these purposes, as a fish with between 8 and 30 plates), which had 25 lateral plates. This partially plated individual will hereafter be termed “intermediate.” We visually inspected a further 1,260 individuals for lateral plate morph in the field across the six lagoons and found only 17 partially plated individuals (1.4%), and the proportion of partially plated fish did not differ between lakes (χ^2^ = 4.26, df = 5, *P *=* *0.5128). In the 239 euthanized individuals, we measured the sizes of a further six elements of body armor (fig. 1*c*) and found them all to be largely correlated, with the first principal component axis (“armor PC1”), explaining 50% of variation in the size of body armor describing an increase in the size of all six armor elements. Armor PC2 (explaining 22% of variation in the size of body armor) described fish with a proportionally larger pelvis and shorter spines. The two ecotypes were largely similar in terms of the relative size of their body armor, although anadromous fish showed slightly increased variability, particularly along armor PC2 (fig. 3*b*). The intermediate individual fell within the 95% confidence ellipses of both anadromous and lagoon resident ecotypes (fig. 3*b*).

Secondly, anadromous ecotypes scored higher, on an average, than lagoon residents along the first principal component axis of a PCA on body shape landmarking coordinates (“shape PC1,” fig. 3*c*, see fig. 1*a* for landmark configurations), which accounted for 29% of total variation in body shape. Anadromous fish exhibited a larger, more pronounced snout, bodies with a deeper anterior and narrower middle section, a thicker caudal peduncle, longer anal fin, more rearward positioning of the pectoral fin and forward positioning of the anal spine (fig. 3*e*). The second principal component (shape PC2) explained 17% of body shape variation but was strongly associated with specimen bending (see supplementary fig. S2, Supplementary Material online, for shape changes associated with shape PC2), a common occurrence in stickleback morphometrics (Wund et al. 2008), and so was not considered further in our analysis. Shape PC3 explained 9% of variation in body shape and largely described increasing overall body depth (fig. 3*f*). Ecotypes were predominantly similar along shape PC3, although anadromous fish were slightly more variable along this axis (fig. 3*c*).

Thirdly, the two ecotypes also differed in overall body size (measured as centroid size: the square root of the sum of the square distances of each landmark to the center of all landmarks) with anadromous fish being considerably larger than lagoon resident fish (*t *=* *34.51, df = 189.9, *P *=* *0.002×10^−^^13^, fig. 3*d*). The intermediately plated individual was also intermediate in body size (although closer to lagoon resident fish; fig. 3*d*).

#### Genetic Differentiation

**Fig. 4.**
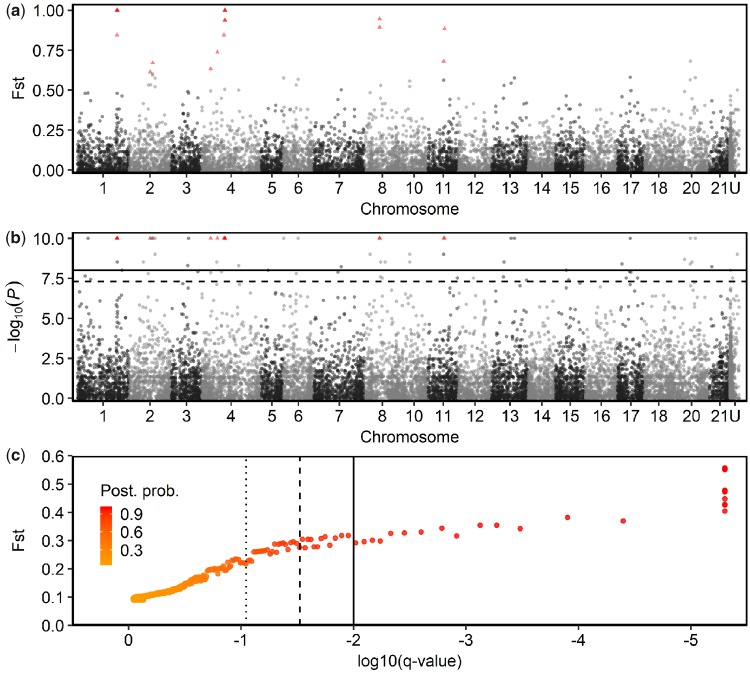
Analyses of outlier SNPs in the Obse species-pair. Manhattan plots showing (*a*) genome-wide Fst estimates for 12,575 SNPs calculated using the POPULATIONS program in the Stacks pipeline, and (*b*) the negative logarithm at base 10 of the *P* values (−log10(*P*)) for the SNPs in (*a*). Horizontal lines in (*b*) represent the 0.05 (dashed line) and 0.01 (solid line) significance threasholds for SNPs under selection. (*a*) and (*b*) show the location of SNPs across the genome, excluding the sex chromosomes. U describes SNPs that mapped to unassigned scaffolds. (*c*) Fst and *q* values (log10) for 12,575 SNPs, estimated using the Bayescan software. Vertical lines mark “strong” (dotted line), “very strong” (dashed line), and “decisive” (solid line) boundaries on Jeffreys’ scale of interpretation, corresponding to posterior probabilities of loci being under selection of 0.91, 0.97, and 0.99, respectively. SNPs identified as decisively under selection in the BayeScan analysis are indicated by red triangles in (*a*) and (*b*). Outlier analyses were based on 34 individuals from one species-pair lake (Obse, see fig. 1*d* for location and supplementary table S1, Supplementary Material online, for lake details).

**Fig. 5.**
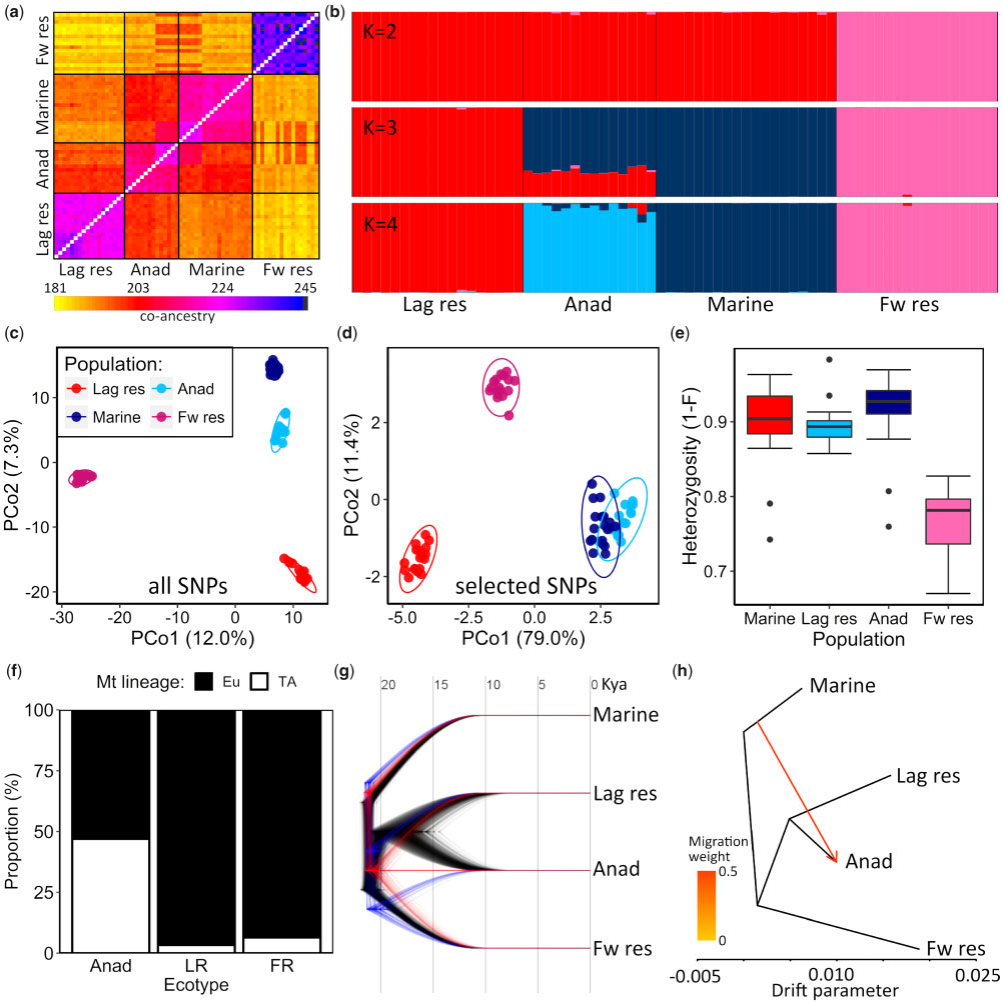
Genomic analyses of a North Uist lagoon resident–anadromous species-pair (Obse) alongside a Uist freshwater resident (Scad) and Icelandic marine population (Nyps) based on genome-wide SNP data. (*a*) Coancestry matrix constructed in fineRADstructure. (*b*) Population structure plots with two (*K* = 2), three (*K* = 3), and four (*K* = 4) inferred clusters output from Structure. Principal coordinate analysis (PCoA) of allele frequencies computed using adegenet for (*c*) all SNPs (*d*) SNPs under putative selection and (*e*) putatively neutral SNPs. (*f*) Proportion of ancient European (Eu) and Trans-Atlantic (TA) mitochondrial lineages across anadromous (Anad), lagoon resident (LR) and freshwater resident (FR) stickleback from North Uist. (*g*) A tree cloud produced using Densitree to visualize the range of alternate topologies of a Bayesian phylogeny produced from a SNAPP analysis in Beast. Divergence time estimates are shown in ka and were calculated using the Icelandic marine population as an outgroup, with an estimated divergence time of 21,100 YBP taken from Fang et al. (2018). Trees shown in black made up 95.8% of concensus tree topologies and trees shown in red and blue made up 2.06% of concensus tree topologies each. Red and blue tree topologies in (*g*) are intensified 2-fold. (*h*) Maximum likelihood tree estimated in TreeMix, with the arrow representing a single migration event that was identified by the optimal TreeMix demographic model. Lag res, lagoon resident; Anad, anadromous; Fw res, freshwater resident.

Genetic analyses from targeted genotyping and genome-wide SNP data strongly suggest that anadromous and lagoon resident ecotypes on Uist are strongly reproductively isolated and maintain genetically distinct genomic regions, despite low levels of gene-flow. First, targeted genotyping at the *Ectodysplasin A* (*Eda*) locus, which has two key alleles; *Eda^L^* (low) and *Eda^C^* (complete), that generally give rise to low- and completely plated phenotypes, respectively (Colosimo et al. 2005), demonstrated that *Eda* is strongly associated with plate morph in Uist species-pairs from Faik, Obse, Duin, Trum, and Dheo (χ^2^ = 81.2, df = 4, *P *=* *0.0022×10^−13^), accounting for 99% of variation in plate number (*n *= 55). Lagoon resident fish (*n* = 33) were fixed for the *Eda^L^* allele and anadromous fish were approaching fixation for the *Eda^C^* allele with only 1 out of the 21 genotyped individuals possessing a heterozygous, rather than homozygous CC genotype (table 1). The only partially plated, intermediate individual sampled across all species-pair lagoons was also an *Eda* heterozygote (table 1), suggesting that the low frequency of partial plate morphs in species-pairs reflects a true low frequency of adult F1 hybrids. Second, outlier analyses on the Obse species-pair suggest that multiple regions of the genome are highly differentiated between anadromous and lagoon resident fish, consistent with evolving under divergent selection. Pairwise Fst computations identified seven fixed differences (SNPs with Fst = 1), four of which fell within a 203,000 bp region within a known chromosomal inversion on chromosome I (Jones, Grabherr, et al. 2012) that contains genes including *atp1a1* and *Igfbp2a*, and three within a 22,500 bp region of chromosome IV containing *Eda* and *Vma21* genes, among others (fig. 4*a*). POPULATIONS identified 50 SNPs as outliers under putative selection (SNPs with associated *P* values <0.01, fig. 4*b*). BayeScan identified 19 SNPs as “decisively” under selection (posterior probabilities >0.99, fig. 4*c*) and all 19 of the outlier SNPs identified by BayeScan were also identified by POPULATIONS. Of these 50 total outlier SNPs, 33 fell within the coding regions of 23 genes (supplementary table S4, Supplementary Material online), including some that have previously been identified as diverging between marine and freshwater populations (Pellissier et al. 2018). Six of the total outlier SNPs fell within the chromosomal inversion on Chr I, which is known to be involved in both marine-freshwater (Jones, Grabherr, et al. 2012) and lake-stream divergence (Roesti et al. 2015). Third, coancestry estimates for the Obse species-pair reveal considerably stronger coancestry within than between ecotypes, with some anadromous individuals from Obse sharing more common ancestry with isolated freshwater resident fish from Scad, an inland lake ∼10 km away (through water), and/or more common ancestry with marine stickleback from Nyps, from the coast of Iceland ∼1,000 km away, than with sympatric saltwater resident individuals in Obse (fig. 5*a*).

**Table 1.** Plate Morph Phenotype Versus Genotype.

Genotype	Plate Morph		
	Low	Partial	Complete
CC	0	0	20
CL	0	1	1
LL	33	0	0

Note.—Lateral plate morph and *Eda* genotype of 55 genotyped individuals from species-pair lagoons on North Uist.

CC, two copies of the *Eda*^C^ allele; LL, two copies of the *Eda*^L^ allele; CL, one copy of *Eda*^C^ and one of *Eda*^L^.

#### Differential Genetic Origins of Species-Pairs

MtDNA sequencing identified 37 composite (cyt *b* + CR) haplotypes across the 76 Uist species-pair individuals (from Faik, Obse, Duin, Trum, and Strm) sequenced in this study, 19 of which are, to the best of our knowledge, previously undescribed. Cyt *b* and control region (CR) sequences were submitted separately to GenBank under accession numbers MG602878–MG602914 and MG602915–MG602951, respectively. We extended our genetic analysis to include 126 individuals from allopatric freshwater populations using sequences taken from Rahn et al. (2016), in an attempt to understand the wider genetic structure and colonization history of Uist stickleback (see fig. 1*d* and supplementary table S1, Supplementary Material online, for sampling locations). Bonferroni corrected pairwise *φST* permutation values indicated significant genetic differentiation in mitochondrial haplotypes between all three ecotypes (anadromous, lagoon resident, and freshwater resident), with anadromous fish being particularly differentiated from the other two ecotypes (table 2).

**Table 2.** Pairwise *φST* and Associated *P* Values for Composite Cyt *b*+ CR mtDNA Haplotypes.

Comparison	*φST*	*P* Value
anad vs. fw res	0.13621	0.00300
anad vs. lag res	0.15065	0.00300
fw res vs. lag res	0.02682	0.02398

Note.—*P* values shown are Bonferroni corrected for multiple comparisons.

Anad, anadromous; lag res, lagoon resident; fw res, freshwater resident.

Bayesian phylogenetic inference on all 202 North Uist mt sequences demonstrated that species-pairs are comprised of two anciently diverged (∼119,000 YBP, although see error margins, supplementary table S5, Supplementary Material online) mitochondrial lineages, which are separated with a posterior probability of 1.00 and correspond to Trans-Atlantic and European lineages identified by Makinen and Merila (2008), supplementary figure S4, Supplementary Material online. We found that the two mitochondrial lineages were present in very different proportions across ecotypes (χ^2^ test: χ^2^ = 49.97, df = 2, *P *<* *0.0001), with the Trans-Atlantic lineage comprising 47% of anadromous, but only 3% and 6% of lagoon resident and freshwater resident populations, respectively (fig. 5*f*). This is consistent with different colonization histories for anadromous and lagoon resident fish, followed by directional maternal introgression from the lagoon resident/freshwater resident populations into the anadromous population. This strongly implies that, rather than originating from a single homogeneous marine population, Uist was colonized by two divergent maternal lineages: one originating in Europe, and the other from further afield in the Atlantic.

#### Admixture and Introgression

To test the hypothesis that species-pairs may have resulted from recent admixture between older populations or lineages, we compared the genetic structure of all four ecotypes that exist on, or have putatively contributed to, stickleback populations on Uist using genome-wide SNP data. This included a species-pair (anadromous and lagoon resident ecotypes) from Obse on Uist, a nearby freshwater resident ecotype from Scad (Uist) and an Atlantic marine population from Nyps in Iceland (as the best available proxy for the second colonizing lineage based on the probable colonization history of Europe; Fang et al. 2018). These four populations were used in all admixture and introgression analyses below (see fig. 1*d* and supplementary table S1, Supplementary Material online, for sampling locations). Multiple analyses of the ancestry of the anadromous–lagoon resident species-pair suggest that anadromous stickleback are genetically admixed between lagoon resident and Atlantic marine stickleback (fig. 5). Firstly, coancestry estimates reveal that anadromous stickleback are genetically more similar to either lagoon resident or marine populations than lagoon resident and marine populations are to one another (fig. 5*a*), indicating that either the anadromous ecotype in Uist species-pairs is of admixed origin, or represents a distinct lineage but with substantial gene flow from both lagoon resident and Atlantic marine populations.

Secondly, with three inferred clusters, Bayesian estimates of population structure and admixture identified the lagoon resident, marine, and freshwater resident populations as unique and genetically distinct, with anadromous fish being admixed and of ∼25% lagoon resident and ∼75% marine ancestry (fig. 5*b*). With four inferred clusters (which was the optimal model with a likelihood of −366,677.5), anadromous fish were treated as a distinct population but with introgression from either marine and/or lagoon resident populations in most individuals (fig. 5*b*). Further increases in the number of clusters resulted almost exclusively in the anadromous population gaining genetic input from alternative unsampled populations (supplementary fig. S5, Supplementary Material online).

Third, principal coordinate analysis (PCoA) of allele frequencies based on all (12,171) SNPs reveal that all four populations form distinct and separate genetic clusters (fig. 5*c*). The primary axis of genetic differentiation (PCo1, explaining 12.0% of allele frequency variation) separates the freshwater from the three saltwater ecotypes. The second axis of genetic variation (PCo2, explaining 5.7% of allele frequency variation) separates the saltwater ecotypes, with marine and lagoon resident ecotypes at either extreme and anadromous individuals falling intermediately, but closer to marine fish, consistent with genetic admixture in the anadromous population. PCoA including only putatively neutral (12,121) SNPs (supplementary fig. S6, Supplementary Material online) was almost identical to the PCoA with all SNPs (fig. 5*c*). PCoA including only the 50 SNPs under putative selection between lagoon resident and anadromous ecotypes (identified by outlier analyses, fig. 4*b* and *c*) lead to a primary axis (PCo1, explaining 79% of allele frequency variation) of differentiation between anadromous and lagoon resident fish (fig. 5*d*). Freshwater resident fish fell intermediately along PCo1 and marine fish fell on top of anadromous individuals, suggesting marine and anadromous populations experience largely similar selection pressures, while freshwater fish do not experience similar selection pressures to the other ecotypes. PCo2 of the PCoA on selected SNPs separated freshwater from the three saltwater ecotypes, further indicating different selection pressures for freshwater individuals.

Fourth, consistent with admixture during the evolution of Uist anadromous fish, inbreeding coefficients suggested that anadromous fish had the highest mean heterozygosity, followed by marine fish, lagoon resident fish, and then freshwater resident fish (fig. 5*e*). Heterozygosity estimates were significantly different between ecotypes (LM: *F*_3,__64_ = 33.842, *P *<* *0.0001), but post hoc pairwise comparisons indicated that this was driven by reduced heterozygosity in freshwater resident fish, compared with the other three ecotypes (table 3 andfig. 5*e*).

**Table 3.** Post Hoc Pairwise *t*-Tests for Population Differences in Heterozygosity.

	Marine	Lag Res	Anad	Fw Res
Marine	—	0.13	0.00	8.29
Lag res	0.90	—	0.00	8.06
Anad	0.65	0.65	—	8.27
Fw res	<0.0001	<0.0001	<0.0001	—

Note.—*T*-statistics are given above the diagonal and *P* values (adjusted for multiple testing using the fdr method) below the diagonal.

Fifth, jacknifed ABBA BABA tests, with populations arranged to test for introgression from the marine population into either lagoon resident or anadromous populations (based on 11,929 SNPs), indicated a significantly positive value of *D*, both with biallelic sites removed (*D* ± SD = 0.1290 ± 0.0027, *P *<* *0.0001) and with random substitution of biallelic sites (*D* ± SD = 0. ± 0.0027, *P *<* *0.0001). This is consistent with an introgression event from marine fish into the anadromous population.

Sixth, Bayesian coalescent-based estimates of the species-tree indicated a largely consistent consensus tree topology (present in 95.88% of 2,000 sampled trees) with anadromous fish diverging recently from lagoon residents (15,000–20,000 YBP, fig. 5*g*), and freshwater resident fish on the island being more anciently derived (∼21,000 YBP). Two alternate tree topologies were identified in the SNAPP runs, indicating low-levels of incomplete lineage sorting, but each was present in just 2.06% of all trees (fig 5*g*). Furthermore, the consensus tree topology is identified by TreeMix, a software designed to construct the maximum likelihood tree of a set of populations and then infer gene-flow based on residual genetic variation not explained by the tree (Pickrell and Pritchard 2012). TreeMix identifies migration from the marine into the anadromous population in the best fitting demographic model, which further supports mixed ancestry of anadromous fish inferred from the analyses above (fig. 5*h*). TreeMix assumes gene flow occurs via discrete migration events (Pickrell and Pritchard 2012), but gene flow may have been long-term and may even be on-going. This is not tested here.

## Discussion

We present compelling morphological and genetic evidence that unusually strong reproductive isolation has developed between lagoon resident and anadromous stickleback in numerous coastal lagoons on the Scottish Island of North Uist, in the face of ongoing low-level gene-flow. Furthermore, we show that the “classic” purely ecologically based speciation model with a recent double-invasion from a single genetically homogeneous marine founder population, driven by RSL change, is unlikely to be responsible for the strong reproductive isolation in Uist stickleback species-pairs. Instead, our results suggest that Uist was colonized by at least two, genetically differentiated stickleback lineages, and are consistent with a role for admixture upon secondary contact providing the genetic substrate needed for phenotypic divergence, alongside strong selection in driving and maintaining phenotypic and genetic differentiation.

### Evidence for Species-Pairs

Sympatrically breeding resident and anadromous stickleback ecotypes are common in coastal regions across much of the Holarctic range of the species, and some morphological and genetic divergence is almost ubiquitous (McKinnon and Rundle 2002). Despite this, persistent admixture occurs to some extent across nearly all documented contact zones (Rafinski et al. 1989; McPhail 1994; Higuchi et al. 1996; Hendry et al. 2009) and reproductive isolation as complete as that which exists on Uist is extremely rare (Bell et al. 2010). The occurrence of low-plated resident and completely plated anadromous morphs in the absence of intermediate partially plated fish occurs in only two known locations: one in Alaska (Karve et al. 2008; Drevecky et al. 2013) and one in Russia (Ziuganov 1995). We identified six locations on Uist in which morphologically intermediate individuals are extremely rare (∼1.4%), and are aware of several others. *Eda* genotyping revealed that *Eda* explains 99% of the variation in plate number in Uist species-pairs, confirming that the rarity of partially plated individuals likely reflects a true absence of adult F1 hybrids rather than being the result of dominance of the *Eda^C^* allele, which has been recorded in a handful of stickleback populations (Cresko et al. 2004; Lucek et al. 2012). Morphological data suggest that species-pairs are largely similar across individual lagoons on North Uist and the close proximity of the lagoons to one another (and their connection by sea) makes it likely that ecotypes across lagoons have a single origin.

Analyses of the species-pair for which SNP data were available (Obse) also implied substantial genetic segregation between sympatric anadromous and lagoon resident ecotypes. All of the methods we used to assess population structure identified the two ecotypes as distinct and separate genetic groups, with very little indication of ongoing admixture. Furthermore, our coancestry estimates showed that some anadromous individuals from the species-pair shared more common ancestry with both an isolated inland freshwater population on Uist that is ∼10 km away through water and an allopatric marine population ∼1,000 km away in Iceland, than they did with sympatric lagoon resident fish, strongly suggesting that the two sympatric ecotypes form two largely independently evolving lineages. Outlier analyses also identified numerous regions of the genome that are under putative selection between lagoon resident and anadromous fish. When mapped back to the annotated genome, many of those regions fall within genes that are of known functional and ecological importance (Colosimo et al. 2005; Jones, Grabherr, et al. 2012; Escudero-Esparza et al. 2013), and some fall within a known inversion on Chr I, which is involved in multiple ecological divergence events in stickleback (Jones, Grabherr, et al. 2012; Roesti et al. 2015), suggesting that genomic divergence between ecotypes is likely to have an ecologically adaptive basis. The results of our Structure analysis indicated that there has been some (low-level) recent introgression from the lagoon resident into the anadromous population, suggesting that this phenotypic and genetic differentiation is maintained despite low levels of gene flow. Bayesian coalescence-based estimates indicated that lagoon resident and anadromous individuals in Obse diverged from one another ∼15,000–20,000 YBP and our optimal TreeMix model of migration implied that there have been no substantial migration events between these two ecotypes since that initial divergence. Taken together, our findings suggest that reproductive isolation in this species-pair is considerably stronger than in most other stickleback examples, and the two ecotypes are coexisting sympatrically with very little gene flow. The evolutionary processes that lead to the formation of species-pairs such as these are complex and still not well understood (Schluter 2009; Richardson et al. 2014), but Uist stickleback populations provide excellent opportunities to shed light on these.

### Origins of Species-Pairs

#### “Classic” Purely Ecological Speciation Model

We found little evidence to support the hypothesis that the evolution of species-pairs on Uist is solely explained by the “classic” double-invasion model of postglacial speciation in fish. This hypothesis states that two colonizations from the same, genetically homogeneous marine population, made as a result of changes in spatial isolation driven by changes in RSL, coupled with ecologically based divergent selection in allopatry, are exclusively responsible for the formation of species-pairs. Applied to Uist species-pairs, this theory made four testable predictions. First that there is evidence for strong, ecologically based selection between ecotypes; second, the species-pairs originated from multiple colonizations from a genetically homogeneous “stock” marine population; third that they are postglacial in age; and fourth, that there was a double-peak in RSL since the retreat of the Pleistocene glaciers allowing a double-invasion with spatial isolation.

Our outlier analysis identified multiple regions of the genome in which strong divergence between lagoon resident and anadromous ecotypes is maintained despite evidence for low-level gene-flow. This suggests these genomic regions are putatively under strong selection between ecotypes. Some of these outlier regions mapped to a chromosomal inversion on Chr I, which is known to be involved in multiple ecological divergence events in stickleback (Jones, Grabherr, et al. 2012; Roesti et al. 2015). Other outliers mapped to genes such as *Eda*, which is associated with lateral-plate morph in stickleback (Colosimo et al. 2005), a known antipredator trait (Bell 2001; MacColl and Aucott 2014) and Igf family genes which are known to be under selection between marine and freshwater environments (Pellissier et al. 2018), suggesting the selection in Uist species-pairs has an ecological basis. This is consistent with “classic” ecological speciation. However, we showed that the species-pairs on Uist are comprised of two anciently diverged mitochondrial lineages, revealing that there were likely multiple colonization events, but they were not made by a single homogeneous lineage of marine fish. While this prior genetic differentiation could be completely unrelated to the evolution of species-pairs, it adds an extra level of variation on which selection can act, and makes it difficult to attribute the evolution of species-pairs entirely to ecological selection for adaptations to the different environments that would have been experienced during a double-invasion.

Our Bayesian divergence time estimates indicated that the species-pairs probably are postglacial in origin, with a divergence time of 15,000–20,000 YBP, which could be consistent with a double-invasion that was driven by changes in RSL. However, this divergence time does not correspond to the timings of any saline influxes in any of the cored species-pair lagoons (which were all considerably more recent), but rather is approximately the same as estimates of the timing of deglaciation of the island (Ballantyne 2010). This is also consistent with speciation being a direct result of secondary contact between older lineages independently colonizing Uist immediately following the glacial retreat. Moreover, our RSL change reconstructions detected an increase in RSL to the present day, which is already well documented for this part of the world (Jordan et al. 2010), but did not detect an earlier period of saline flooding in any of the three species-pair lakes for which reconstructions were made. While it is possible that the basal sediment in our cores (∼13,000 YBP for the radiocarbon dated core) did not extend to the beginning of the interglacial period on the island and we missed an earlier saline “high-stand” period, or that the speciation event occurred in another lake that was of a different elevation and did experience an earlier saline influx with subsequent migration of the divergent ecotypes into the lakes that we sampled, our alternative explanations are more parsimonious.

#### Colonization by Prediverged Lineages

We also hypothesized that Uist may have been colonized by lineages that were already reproductively isolated prior to colonization and that secondary contact, with or without admixture could be responsible for the evolution of species-pairs on North Uist. These hypotheses predicted that Uist was colonized by multiple prediverged lineages, there was a differential genetic origin of ecotypes, the prior genetic divergence existed before the colonization of Uist (and thus must predate the retreat of the Pleistocene glaciers on Uist), and either there was no evidence for recent genetic admixture, or one or both of the species-pair ecotypes is genetically admixed in relation to putative parental populations.

Our analyses confirmed that Uist is a meeting place for two predominantly allopatric, ancient mitochondrial lineages: the Trans-Atlantic and European lineages (Makinen and Merila 2008), which diverged ∼119,000 YBP, long before the most recent glaciers on Uist would have melted (Ballantyne 2010). We also showed that these lineages occur in very different proportions in different ecotypes, with resident stickleback being almost entirely of European origin, but anadromous fish being an approximately equal mix of the two. There are at least two explanations for this pattern. First, is could suggest that resident and anadromous ecotypes were independently founded by the European and trans-Atlantic lineages, respectively and experienced some (mostly unidirectional) introgression upon initial secondary contact, implicating admixture in speciation. In this case, introgression must have been almost ubiquitously between lagoon resident females (with Eu mtDNA) and anadromous males, a pairing which seems much more likely than anadromous females (with TA mtDNA) mating with lagoon resident males, given that lagoon resident fish are considerably smaller than anadromous fish. Alternatively, Uist may have initially been colonized by the European lineage, which gave rise to both resident and anadromous populations, and fish of trans-Atlantic origin arrived later, and failed to introgress into resident populations. In the latter case, it is possible that barriers to gene flow between the two ecotypes already existed, preventing trans-Atlantic mitochondrial haplotypes from entering resident populations.

For a number of reasons, however, it is more likely that the primary reason for the lack of trans-Atlantic haplotypes in resident populations is that anadromous fish carrying trans-Atlantic mitochondrial haplotypes are less well adapted to a resident lifestyle. Firstly, Anadromous fish of trans-Atlantic origin in Europe are descendant from fish which must have crossed the Atlantic, and therefore probably possess a greater suite of adaptations to an oceanic rather than a resident existence. Fish in Europe with European mitochondrial haplotypes, on the other hand, have probably spent much more of their recent evolutionary history as resident populations, making them likely to be better adapted to a resident lifestyle. Second, there are locations on Uist in which freshwater resident and anadromous stickleback do hybridize (MacColl A.D.C., Dean, L.L. and Whiting, J.R., unpublished data), which should result in trans-Atlantic mitochondrial haplotypes infiltrating freshwater resident populations, and yet this largely appears not to be the case. Interestingly, our outlier analysis identified six SNPs (by far the most within any one gene) within the vacuolar H+-ATPase (*Vma21*) gene as being under strong selection between lagoon resident and anadromous fish. The function of *Vma21* is likely related to ATP synthesis (Finbow and Harrison 1997), a pathway which also involves many mitochondrially encoded proteins, and thus perhaps mitonuclear conflict/incompatibilities have played a part in the evolution of species-pairs. Further investigations would be necessary to draw conclusions about this exciting possibility. Regardless of the mechanism, genetic differences between lineages could thus have been involved in the formation of species-pairs.

#### Admixture

MtDNA sequencing revealed that the anadromous population on Uist is comprised of two ancient maternal lineages, which occur in approximately equal proportions, suggesting that admixture has been particularly important in the evolution of the anadromous ecotype on Uist. MtDNA, however, only relays information about the maternal line, and thus we also compared the autosomal DNA of a species-pair with that of local freshwater resident and Icelandic marine (as a proxy for an Atlantic marine founder population) stickleback to investigate the genetic relationships between ecotypes. Our analyses of the autosomal genome-wide SNP set indicated that the anadromous population on Uist is genetically admixed, with genomic input from lagoon resident (the other half of the species-pair) and Atlantic marine populations. Genetic admixture can provide novel combinations of genes on which selection can act, and is most likely to be involved in speciation when recombinant phenotypes are better adapted to a given niche than either parental species, allowing admixed individuals to exploit environments that are unavailable to either parent species (Schumer et al. 2014). We therefore hypothesize that an initial marine colonization event may have given rise to freshwater and lagoon resident ecotypes on Uist as in many other parts of the world (McKinnon and Rundle 2002). Then the proximity of Uist to the Atlantic lead to subsequent admixture between these derived ecotypes and the fully marine stickleback population in the Atlantic, about which very little is known (Ahnelt 2018). Admixture would likely have produced some individuals with a combination of adaptations to an oceanic lifestyle, but also a propensity to spawn in the safety of coastal regions, a combination not found in either parental population, that could allow them to simultaneously exploit both environments as anadromous fish. The habit of migrating to sea in anadromous fish could be enough by itself to cause strong disruptive selection from lagoon resident fish. Hybrids with a tendency to migrate, but without the full genetic physiological or antipredator “toolkit” to live in the sea, would fall in a valley of very low fitness and be unlikely to reach adulthood.

In a phylogeny of Uist stickleback populations (with Icelandic marine fish as the outgroup), Uist anadromous fish do not approximate the marine founders of the island, as would be expected by traditional models of stickleback dispersal (Colosimo et al. 2005; Schluter and Conte 2009). Rather, freshwater resident stickleback fall as the outgroup to other Uist populations, and the anadromous population evolved more recently from lagoon resident fish. By modeling historic migration events, we were able to show that anadromous fish received genetic input from the Icelandic marine population (our proxy for Atlantic marine stickleback) during their divergence from lagoon resident fish. Whether or not this could be defined as “hybrid speciation” depends on how the term is defined (Schumer et al. 2018), but our findings add to the growing body of evidence suggesting that speciation is not a linear, bifurcating process, but is in fact far more reticulate than was once widely thought (Martin-Bravo et al. 2010; Frantz et al. 2013; Alexander et al. 2015), with admixture events often playing a key role in the process (Mallet 2007; Comeault and Matute 2018; Marques et al. 2019).

## Conclusions

We have identified unusually strong reproductive isolation between sympatric anadromous and lagoon resident stickleback ecotypes, in multiple lagoons on the Scottish Hebridean island of North Uist. We tested three hypotheses, which were not mutually exclusive, to explain how such strong reproductive isolation has evolved. While we cannot completely rule out contributions from any of our three models, our results indicate that the “classic” explanation for more pronounced reproductive isolation in stickleback, an ecological speciation model driven by postglacial changes in RSL, is unlikely to be responsible for speciation in the present case. Instead, our results suggest that the most parsimonious explanation probably involves genetic admixture upon secondary contact between multiple colonizing lineages/ecotypes that provided the basis for strong (at least partially ecologically based) divergent selection. These findings are in line with much recent research that is beginning to suggest that cases of seemingly recent, purely ecologically based speciation are actually cryptic examples of speciation that has a much older genetic basis, that developed allopatrically (Bernatchez and Dodson 1990; Feder et al. 2003; Kuehne et al. 2007; Foote and Morin 2015; Foote 2018; Marques et al. 2019). This study demonstrates that proper interdisciplinary investigations of localized geographical changes should be made before those changes can be assumed to have driven speciation via changes in habitat connectivity and population range shifts, particularly with regards to geographical events that can be highly variable across small spatial scales, such as RSL change. Our study also highlights how genetic data can be used to test historic demographic hypotheses and demonstrates that an interdisciplinary approach, combining genetic, morphological, and geographical data are likely to give the most complete picture of historic speciation events. Finally, we have identified a new system which provides an exciting future opportunity to investigate parallelism across species-pairs in multiple lagoons.

## Materials and Methods

### Sampling Design

We collected sediment core sequences for RSL change reconstructions from species-pairs lagoons during two field trips to Uist in 2013 and 2015 (supplementary section 1, Supplementary Material online). We collected stickleback from species-pair lagoons during spring of 2015 for morphological and genetic analyses (supplementary section 2, Supplementary Material online). We also obtained an additional RAD-seq SNP data set including 70 individuals from three populations on Uist and one in Iceland from Magalhaes et al. (2016) and mitochondrial sequences from a further 126 Uist stickleback from Rahn et al. (2016) to extend our understanding of where Uist species-pairs fit within the wider radiation of stickleback, and to identify their origins.

### RSL Change Reconstructions

Briefly, to reconstruct changes in RSL on Uist over the Holocene period, we precisely mapped the elevation of three species-pair lagoons, collected long sediment sequences from them, and predicted past changes in salinity using a discriminant function trained with modern Uist sediment samples. We then radiocarbon dated ancient sediment samples to date marine—freshwater/freshwater—marine transitions. For details, see supplementary section 1 of the Supplementary Material online.

### Morphological Analyses

To quantify morphological differentiation, stickleback were sampled from six species-pair lakes on Uist: Faik, Obse, Duin, Trun, Strm, and Dheo (see fig. 1*d* for lake locations and supplementary table S1, Supplementary Material online, for detailed sampling information). We measured morphological differences in species-pairs by quantifying differences in three key aspects of morphology: body size, body shape, and external body armor. Briefly, individuals were stained to highlight external skeletal structures before measurements of various aspects of body armor were taken. We then used a geometric morphometric landmarking approach to measure differences in body shape and body size. For details, see supplementary section 3 of the Supplementary Material online.

### Genetic Analyses

To ascertain whether morphological differences in lateral plate morph reflected underlying genetic segregation in species-pairs, we genotyped a subset of individuals (from Dheo, Duin, Faik, Obse, and Trum, see fig. 1*d* for lake locations and supplementary table S1, Supplementary Material online, for detailed sampling information) at the *Eda* locus, which is involved in determining lateral plate phenotype (Colosimo et al. 2005), and made genotype–phenotype comparisons (see supplementary section 4a of the Supplementary Material online for details). We constructed various SNP data sets from those published in Magalhaes et al. (2016) and SNPs from an Icelandic population (Nyps), which was sequenced and processed at the same time. Our SNP data sets were constructed to include different individuals, populations, and filtering for different analyses (for a detailed description of SNP data sets, see table 4). To identify regions of the genome under putative selection in a species-pair (Obse), we used POPULATIONS in the Stacks pipeline (Catchen et al. 2013) and BayeScan version 2.1 (Foll and Gaggiotti 2008), which were run on SNP data set 1 (see table 4 for details). For further details about SNP analyses, see supplementary section 4b, Supplementary Material online.

**Table 4.** SNP Data Sets Used in Population Genetic Analyses.

Data Set	*N* ind	*N* SNPs	Lake/Ecotype/*n*	LD Thinned	Sibs Removed	Neutral SNPs	Selected SNPs
Data Set 1	34	12,575	Obse: anad (16) Obse: lag res (18)	×	×	✓	✓
Data Set 2	68	11,930	Obse: anad (14) Obse: lag res (18) Nyps: marine (19) Scad: fw res (17)	×	✓	✓	✓
Data Set 3	68	9,464	Obse: anad (14) Obse: lag res (18) Nyps: marine (19) Scad: fw res (17)	✓	✓	✓	✓
Data Set 4	70	12,171	Obse: anad (16) Obse: lag res (18) Nyps: marine (19) Scad: fw res (17)	×	×	✓	✓
Data Set 4a	70	50	Obse: anad (16) Obse: lag res (18) Nyps: marine (19) Scad: fw res (17)	×	×	×	✓
Data Set 4b	70	12,121	Obse: anad (16) Obse: lag res (18) Nyps: marine (19) Scad: fw res (17)	×	×	✓	×
Data Set 5	70	9,464	Obse: anad (16) Obse: lag res (18) Nyps: marine (19) Scad: fw res (17)	✓	×	✓	✓
Data Set 5a	23	1,000	Obse: anad (3) Obse: lag res (9) Nyps: marine (4) Scad: fw res (7)	✓	×	✓	✓
Data Set 5b	23	1,000	Obse: anad (4) Obse: lag res (10) Nyps: marine (5) Scad: fw res (4)	✓	×	✓	✓

*N* ind, number of individuals; *N* SNPs, number of SNPs; *n*, sample size; LD thinned, whether or not SNPs were thinned to 2,000+bp apart to account for linkage disequilibrium; Sibs removed, whether or not siblings were removed; neutral SNPs, whether or not neutral SNPs were included; selected SNPs, whether or not selected SNPs were included.

To investigate whether North Uist is a meeting place for multiple ancient mitochondrial lineages, we sequenced 76 species-pair individuals collected from five lagoons (Faik, Obse, Duin, Trum, and Strm, See supplementary table S1, Supplementary Material online, for detailed sampling information) for two mitochondrial regions: the cytochrome *b* (cyt *b*) gene and a partial fragment of the D-loop CR, which are known to resolve ancient mitochondrial lineages present in the Atlantic and the seas around Europe (Makinen and Merila 2008). We obtained a further 126 concatenated sequences from Uist stickleback from Rahn et al. (2016) and aligned them with our own, resulting in a final 1,380 bp alignment of 202 Uist individuals. To determine whether the genetic structure in Uist mtDNA sequences corresponded to the ancient mitochondrial lineages identified by Makinen and Merila (2008), we collapsed our individual sequence data into haplotypes and constructed a Bayesian phylogeny including the haplotype sequences published in Makinen and Merila (2008), downloaded from Genbank, using MrBayes version 3.2.2 (Ronquist and Huelsenbeck 2003). We then estimated divergence times between the two lineages that were identified in Uist stickleback using coalescence-based MCMC simulations implemented in IMa2 (Hey and Nielsen 2004) to ensure that our divergence times were approximately similar to those in Makinen and Merila (2008). For further details of all mitochondrial analyses, see supplementary section 4c, Supplementary Material online.

To test the hypothesis that admixture may have been important in the evolution of species-pairs, we conducted a variety of analyses using the genome-wide SNP data (table 4). We attempted to compare all current ecotypes present on Uist alongside those which probably resemble the islands marine colonizers. To that end, we compared lagoon resident and anadromous individuals from a species-pair (Obse) with those from a nearby (∼10 km through water), but isolated freshwater population on Uist (Scad) and individuals from a marine population in Iceland (Nyps, ∼1,000 km away). The Icelandic marine population was used as the best available proxy for oceanic stickleback in the Atlantic since marine stickleback in Iceland likely approximate the ancestral Atlantic colonizers of Uist (Fang et al. 2018). First, to investigate the extent of shared coancestry between populations, we constructed a coancestry matrix using data set 2 (see table 4 for details) in fineRADstructure version 0.3.1 (Malinsky et al. 2018). Second, we used data set 3 (table 4) to estimate genetic structure and the optimal number of genetic clusters across populations (models with one to six clusters were tested) in Structure version 2.3.4 (Pritchard et al. 2000; Falush et al. 2003). Third, we conducted a PCoA using the *adegenet* (Jombart 2008) package in R version 3.4.4 (R.Core.Team 2017) on data set 4 (including all SNPs), data set 4a (including only SNPs determined to be under selection in outlier analyses above) and data set 4b (including only SNPs determined to be evolving neutrally in outlier analyses above, table 4) to assess the relative positions of populations in multidimensional genetic space, both overall, and in terms of shared or different selection pressures. Fourth, to investigate heterozygosity, we used data set 4 to estimate inbreeding coefficients (F) on a per-individual basis in VCFtools version 0.1.16 (Danecek et al. 2011). Fifth, to identify introgression from Nyps into the species-pair, we used ABBA BABA tests on data set 2 to estimate jacknifed *D* statistics, both with biallelic sites removed and with random substitutions of biallelic sites using custom R scripts (see supplementary section 4b, Supplementary Material online, for access to R scripts). Sixth, to investigate the colonization history of Uist, we estimated multilocus phylogenetic trees and population divergence times using data sets 5a and 5b in SNAPP analyses (Bryant et al. 2012), implemented in Beast version 2.5.1 (Drummond and Rambaut 2007). Finally, we constructed a maximum likelihood tree for the same populations and modeled historic migration events using data set 5 in TreeMix version 1.13 (Pickrell and Pritchard 2012). For further details of all SNP analyses, see supplementary section 4b of the Supplementary Material online.

## Supplementary Material

Supplementary data are available at *Molecular Biology and Evolution* online.

## Supplementary Material

msz161_Supplementary_Data
