# Supplementary material for: Admixture between Ancient Lineages, Selection, and the Formation of Sympatric Stickleback Species-Pairs
Source: Mol Biol Evol. 2019 Jul 16;36(11):2481–97. doi: 10.1093/molbev/msz161 (PMC6805233; doi:10.1093/molbev/msz161)
Supplement: msz161_Supplementary_Data [file msz161_supplementary_data.zip › Supplementary material_revised.pdf]

Supporting information

Section 1: Relative sea-level (RSL) change reconstructions

Three species-pair lagoons; Obse, Faik and Strm (Figure 1d, see Table S1 for detailed sample site information), were selected for marine inundation history reconstructions. To identify the lowest point at which sea water could enter each lagoon, the precise elevation (±0.05m) of the underlying rock sills connecting each lagoon to the sea was mapped using detailed digital terrain modelling via Real-Time Kinematic (RTK) Global Navigation Satellite Systems (GNSS) corroborated with Benbecula OS active station GNSS data.

To reconstruct Holocene saline influxes, long sediment sequences were extracted from Obse, Faik and Strm in spring of 2013 and 2015. A further five surface sediment sequences of at least 20cm were collected from two freshwater (Iala and Crei) and three saline (Bhoi, Dheo and Port) basins on North Uist (see Figure 1d for locations and Table S1 for detailed sampling information) in spring 2015, to accurately characterise present freshwater and saline conditions. Sediment sequences were collected using either a 1.5m Livingston style piston corer (7cm diameter) for deeper sediments or a Glew gravity corer (10cm diameter) for surface sediments suspended from a boat raft. For long cores, successively deeper 1m drives with 20cm overlaps were taken until hard basal sediment or bedrock was reached. Cores were retrieved from the deepest part of each basin, located using a handheld echo sounder. Short cores were extruded and sectioned into 1cm intervals in the field. Long sediment sequences were extruded into half drainpipes and wrapped in cling film in the field, after which they were stored at ~5°C until sectioning approximately one month later.

Following stratigraphic description, long cores were sectioned into 1cm intervals and stored at ~5°C in sealed plastic bags. Samples for energy dispersive X-ray fluorescence (ED-XRF) were air-dried, ground and homogenised prior to analysis. ED-XRF measurements (ppm) of Na, Si, S, Cl, K, Ca, Cu, Zn, Br and Sr, elements with known salinity associations (Ziegler et al. 2008; Chague-Goff et al. 2016; Filikci et al. 2017), were recorded at 5cm intervals for long cores and 2cm intervals up to

a depth of 19cm for surface cores using a PANalytical Epsilon 3 XLE benchtop ERF energy fluorescence spectrometer. A linear discriminant analysis (LDA) was performed to create a discriminant function using the (scaled) concentrations of all measured elements from the top 19cm of surface sediments from short cores (deposited under known salinity conditions) as a calibration series, with 'saline' (<20,000 μS/cm) and 'freshwater' (<250 μS/cm) as grouping variables using the MASS package, version 7.3-45 (Venables and Ripley 2002) in R version 35.2 (R.Core.Team 2017). The resulting discriminant function (Figure 2a) was used to predict historic salinity for long core sediment (Figure 2b). The accuracy of the LDA in correctly predicting group membership of sediment samples within the training set was determined using a jack-knifed, leave-one-out cross validation approach and the accuracy of long core predictions was determined using maximum posterior probabilities.

To corroborate elemental-based salinity estimates, samples from Obse were extracted and prepared for diatom analysis following standard procedure (Battarbee et al. 2001). Diatoms (>300 valves per slide) were counted and identified following the nomenclature in (Krammer and Lange-Bertalot 1988c, a, b) and Snoeijs (1993) to effectively match the nomenclature used by Van Dam et al. (1994) for salinity tolerance classifications (Table S2). Diatom taxa were classified according to their salinity tolerances following Van Dam et al. (1994).

Six radiocarbon dates were obtained from areas of interest in the Faik long core (Table S3). Plant macrofossil samples were prepared to graphite by the NERC Radiocarbon Facility (East Kilbride) and dated by the SUERC (Scottish University Environmental Research Centre) AMS (accelerated mass spectrometry) Laboratory. Calibrations were made using CalPal (Weninger et al. 2007). The accumulation history of sediment deposits in Faik was estimated using a Bayesian age-depth model based on the six radiocarbon dates (Table S3), implemented using the package rbacon version 2.3.7 (Blaauw and Andres Christen 2011) in R version 3.5.2 (R.Core.Team 2017), and the model was used to predict the age of each cm of sediment in the Faik long core. The linear sedimentation rate in Faik was calculated by dividing the basal radiocarbon date by the basal depth

and its consistency over time was assessed by plotting age-depth correlations for all six radiocarbon dates (Figure S1). Sedimentation rates were likely to be similar across lagoons because of their close proximity and similar attributes, therefore the age of sediment deposits in Obse and Strm long cores were estimated by transposing the mean Faik sedimentation rate to these cores.

Section 2: Stickleback sample collection

To characterize morphological and genetic separation between previously undescribed sympatric lagoon resident and anadromous species-pairs, we collected between four and 42 individuals per ecotype (lagoon resident and anadromous), per lagoon, along with all morphologically intermediate individuals (this was only one individual across all lagoons) from six coastal lagoons on North Uist: Faik, Obse, Duin, Trum Strm and Dheo (see Figure 1d for locations and Table S1 for detailed sampling information) between April and May of 2013 and 2015. Captured sampled stickleback were immediately euthanized with an overdose of tricaine methanesulfonate ('MS222') (400 mg L^{-1}), and killed by destruction of the brain, in accordance with Schedule One of UK Home Office regulations. The caudal fin and both pectoral fins were removed from dead fish and stored in 100% ethanol at -20°C for genetic analyses. Finally, fish were preserved in 70% ethanol prior to morphometric analysis. To crudely survey the proportion of morphologically intermediate individuals in species-pairs we sampled a further 1021 individuals across the six lagoons (giving a total sample size of 1260) during spring of 2018, and recorded whether they were low (3-7), partial (9-28) or completely plated (30-33) before returning them to their site of capture, unless they were required for other analyses. All fish were collected using eight to thirty un-baited minnow traps (Gee traps, Dynamic Aqua, Vancouver) per lake set overnight in water approximately 30-100cm deep.

Section 3: Morphological analyses

To visualize external bony 'armour' structures all individuals collected from six species-pairs (n=239) were bleached and stained with alizarin red following standard procedure (Peichel et al. 2001). The left side of each stickleback was photographed using a tripod mounted digital SLR camera

fitted with a macro lens and macro digital ring flash. Images were scaled and counts of lateral plate number, alongside measurements of standard length, first and second dorsal spine length, longest plate length, pelvis height, pelvis length and pelvic spine length were taken (continuous elements to the nearest 0.01mm) using ImageJ, version 1.52a (Schneider et al. 2012), Figure 1b and 1c. All continuous armour variables (thus excluding plate number, which was independent of body size in our data set and is considered separately here, Figure 3a) were size-standardized by taking the residuals of a regression against standard length. To visualize the axis of greatest variation in bony armour, a PCA was carried out using the singular value decomposition method with scaling on the armour regression residuals (Figure 3b). To test for between-lake differences in the proportions of partially plated fish a chi-squared test was used with lake (Faik, Obse, Strm, Duin, Trum and Dheo) and plate-morph (low, partial and complete) as tabulated variables. To determine whether the number of lateral plates differed between anadromous and lagoon resident ecotypes a t-test was used. All analyses, unless otherwise stated, were performed in R, version 3.5.2.

To analyse body shape, images were re-scaled and 27 landmarks, based on configurations which have previously been shown to sufficiently describe stickleback morphometrics (Walker and Bell 2000), were placed on each image using tpsDig, version 2.16 (Rohlf 2010), Figure 1a. Landmark data were analysed using MorphoJ, version 1.06d (Klingenberg 2011). A Procrustes fit, aligning specimens by their main axis, was performed to remove size and rotation bias from landmark configurations and a Procrustes ANOVA with ecotype (anadromous, lagoon resident and intermediate) as the classifier was performed to identify between-group differences in size and shape. Allometric variation in body shape was removed using a multivariate partial least squares regression analysis, with log centroid size as the independent variable and the regression was pooled within ecotype covariances because the Procrustes ANOVA indicated differences between group centroids (Reist 1986). Regression residuals were then exported into R where they were standardised and scaled, and variation in body shape across all individuals was visualized using a principal components analysis (PCA, Figure 3c), implemented by singular value decomposition.

To assess differences in body size the centroid size (Figure 3d) of each individual was calculated from the 27 landmark configuration used to measure body shape (Figure 1a) in MorphoJ. Centroid size measurements were exported into R and differences in body size between anadromous and lagoon resident ecotypes were evaluated using a t-test.

Section 4: Genetic analyses

Section 4a: Eda genotyping

Eda genotyping was used as a simple assay of the occurrence of adult F1 anadromous – lagoon resident hybrids given that, based on phenotype, the parental populations are expected to be homozygous for alternate alleles (Table 1). Genomic DNA was extracted from fin clips for a subset of sampled fish in species-pairs (33 resident, 32 anadromous and the single morphologically intermediate stickleback) across five of the six species-pair lakes (excluding Strm) using either Qiagen DNeasy blood and tissue kits, Quanta Biosciences Extracta™ DNA prep for PCR-Tissue kits or following a proteinase K and ethanol precipitation procedure (Goldenberger et al. 1995). DNA was amplified for the *Stn382* microsatellite marker, which flanks a 60bp indel in the first intron of *Eda,* using the primers: forward 5' CCCTTAGAGAATTTCCTAGCAG 3', reverse 5' CTTGTCCCGGATCATACGC 3', taken from (Colosimo et al. 2005). Polymerase chain reaction (PCR) was carried out in 20µl reaction volumes consisting of 8µl nuclease-free H_20, 9µL 2X Biomix™ red reaction mix (Bioline), 1µl of forward and reverse primers (10µM) and 1µl of template DNA (approximately 20ng). Thermocycling was carried out as follows: Initial denaturation at 98°C for two minutes, followed by 35 cycles of denaturation at 98°C for 15 seconds, annealing at 58°C for 15 seconds and extension at 72°C for 30 seconds, followed by a final extension at 72°C for five minutes. *Stn382* produces either a 158bp product, associated with the Eda^L allele or a 218bp product associated with the Eda^C allele (Colosimo et al. 2005). PCR products were analysed on a 1.5% agarose gel in TE buffer at 110V for 50 minutes and product size was determined by visual comparison with 100bp ladder. The effect of *Eda* genotype on plate morph was assessed using a chi-squared test with

‘complete’, ‘partial’ and ‘low’ plated categories for plate morph and ‘CC’, ‘CL’ or ‘LL’ genotypes. The proportion of variance in plate number that was explained by *Eda* genotype was calculated using McFadden’s pseudo R^2 (McFadden 1973).

Section 4b: Genome-wide SNP analysis

To further assess genetic differentiation in species-pairs and to test hypotheses about their ancestry, geonome-wide SNP data were acquired for 16-19 individuals per population, from four populations in three lakes: both ecotypes of one lagoon resident - anadromous species-pair (Obse), an isolated freshwater population on North Uist (Scad) and an Icelandic marine population (Nyps, see Table S1 for lake details). All SNP data was acquired as BAM files from those published in Magalhaes et al. (2016), or those processed at the same time in the same way, from genomic DNA collected in 2013 and 2014. Several datasets were created to include different population combinations and site filtering for different analyses (Table 3). In all cases, BAM files were called into a single VCF file using the Stacks pipeline (Catchen et al. 2013) and POPULATIONS program in Stacks. The pipeline was run with the following filters for all data sets: SNPs with a minimum depth of coverage < 3 were removed; SNPs present in < 80% of individuals within a population were removed; SNPs with a minor allele frequency < 0.05 were removed; SNPs that were not present in all of the populations were removed; and only the first SNP of each RAD locus was retained to avoid linked loci. All datasets were further filtered using VCFtools version 0.1.16 (Danecek et al. 2011) to remove sites with mean depth values (over all individuals) < 6 and > 200, sites with > 25% missing data, sites with a minor allele count over all individuals < 2 and the sex chromosomes. Initial inspection of sequences indicated that two anadromous individuals from Obse had extremely high sequence homology. Calculations of pairwise unadjusted Ajk relatedness estimates (Yang et al. 2010), made using VCFtools, confirmed that these individuals had an extremely high Ajk value (1.09768), and were probably siblings. These two individuals were removed in some data sets to avoid their high sequence similarity obscuring other information in the outputs. Some datasets were thinned to remove loci within 2000bp of one another to remove possible effects of linkage

disequilibrium (see below for justification of linkage filtering). Individual dataset filtering options and attributes are given in Table 3 and all further filtering was carried out using VCFtools and BCFtools version 1.9 (Li et al. 2009).

To detect signatures of selection between anadromous and lagoon resident populations we used two methods for detecting outlier loci, implemented using dataset 1 (Table 3). Firstly, pairwise Fst (Figure 4a) and their associated *p*-values (calculated using Fisher's Exact Test) were computed using the POPULATIONS program in Stacks. SNPs with *p* values falling below a conservative 0.01 significance threshold were identified as outliers (Figure 4b). Secondly, a reversible jump Markov Chain Monte Carlo method based on estimations of the posterior probabilities of loci being under selection was implemented in BayeScan, version 2.1 (Foll and Gaggiotti 2008). The dataset 1 VCF file was converted to BayeScan format using PGDSpider version 2.1.1.5 (Lischer and Excoffier 2012). The BayeScan analysis was run with 20 pilot runs of 5000 iterations, a burnin of 50000 iterations and a further 100000 iterations and prior odds for the neutral model were set to 10. BayeScan defines several levels of outlier detection based on Jeffreys' scale of interpretation with 'strong', 'very strong' and 'decisive' margins as evidence for selection, which correspond to posterior probabilities of loci being under selection of 0.91, 0.97 and 0.99 respectively. In the interests of conservativism we determine outlier loci to be those which fall above 'decisive' evidence (posterior probability >0.99) for selection (Figure 4c). The functional importance of all identified outlier loci (Table S4) was assessed by mapping back to the annotated reference genome on Ensembl genome browser, version 94.

To determine co-ancestry across populations we constructed a co-ancestry matrix (Figure 5a) using dataset 2 and the program fineRADstructure (Malinsky et al. 2018). The RADpainter tool is efficient at estimating the effective number of loci in mapped data files and so prior filtering for linkage disequilibrium was not necessary. Siblings were removed in this analysis as their inclusion altered the scale on which ancestry was plotted and obscured relationships between other individuals and populations. The fineSTRUCTURE (Lawson et al. 2012) clustering algorithm was run with a

burnin of 100,000 steps followed by 100,000 sampled iterations and the tree building algorithm was run with a burnin of 10,000.

To determine population structure we used dataset 3 in Structure version 2.3.4 (Pritchard et al. 2000; Falush et al. 2003). SNPs were thinned to fully account for linkage disequilibrium and siblings were removed to avoid their extreme relatedness identifying them as an independent cluster in Structure. Five independent runs were carried out for K = 1 to K = 6. Each run was performed with a different random number starting seed using an admixture model with a burnin period of 50,000 iterations followed by 500,000 MCMC steps. All other parameters were set to their default values. Convergence and adequate mixing of the MCMC iterations was confirmed using CLUMPP (Jakobsson and Rosenberg 2007) and structure plots for each value of K with the highest likelihood across the five runs are shown (Figure 5b and Figure S4).

To determine the extent of genetic similarities and similarities in selection pressures between populations we conducted principal coordinate analyses (PCoA) using dataset 4 (including all SNPs, Figure 5c), dataset 4a (including SNPs putatively under selection in species-pairs, figure 5e) and dataset 4b (including SNPs that are putatively neutral in species-pairs, Figure 5d). Putatively neutral and selected SNPs were identified in POPULATIONS and BayeScan analyses above. Siblings were included in the datasets for these analyses as their inclusion would not be likely to cause any bias or obscurity in the output. All PCoA were performed using Elucidean distances with the package *adegenet* (Jombart 2008) in R version 3.5.2 (R.Core.Team 2017).

To estimate heterozygosity we calculated the inbreeding coefficient (F) on a per-individual basis using dataset 4 and the --het flag in VCFtools. To test whether there were significant differences in heterozygosity between populations we used a linear model with population (marine, anadromous, freshwater resident and lagoon resident) as the predictor variable and the inbreeding coefficient (F) as the response variable. To determine which populations were driving the significance of the

population term in the linear model post-hoc pairwise t-tests were used. All combinations of populations were tested and *p*-values were adjusted for multiple testing using the fdr method.

To estimate introgression we used 'ABBA BABA' comparisons (Durand et al. 2011) to calculate *D* using a custom R script based on the CalcD function in the evobiR package (Blackmon et al. 2015). Custom R scripts are available on GitHub (https://github.com/lldean18/R_scripts). *D* statistics were calculated both with biallelic sites removed and with biallelic sites randomly substituted using the dataset 2 VCF file, which was converted to FASTA format using PGDSpider. Populations were ordered such that introgression between Obse or Obsm and Nyps would be detected with the freshwater population Scad (which is allopatric and thus highly unlikely to be introgressed with the other three populations) as an outgroup. P-values were computed using a blocked jacknifing approach with blocks of 100 SNPs and 1000 permutations.

To estimate multi-locus phylogenetic trees we used Bayesian coalescent-based methods in SNAPP version 1.1.5 (Bryant et al. 2012) implemented in BEAST version 2.5.1 (Drummond and Rambaut 2007). Because of the high computational demands of running SNAPP and the fact that SNAPP can accurately resolve species trees with n=1 per species (Bryant et al. 2012) we created two datasets (dataset 5a and dataset 5b) with a random sample of approximately 1/3 of the total number of individuals across the four populations in dataset 5 (Table 3) using a custom bash script available from GitHub (https://github.com/lldean18/Bash_scripts/blob/master/make_SNAPP_analysis.sh). We further filtered datasets 5a and 5b such that only 1000 randomly selected SNPs were retained (to increase computational efficiency) and prepared the xml input files for SNAPP using a custom version of the Ruby script snapp_prep.rb (https://github.com/mmatschiner/snapp_prep/blob/master/snapp_prep.rb). The starting tree was set to: (NYPS,(OBSM,(OBSE,SCAD))); based on the assumption that the Icelandic marine population (Nyps) would form the outgroup to North Uist populations and the anadromous population on North Uist would represent the marine ancestor of lagoon and freshwater resident populations as is thought to be the case in North Uist stickleback (Haenel et al. 2019). The divergence time for the outgroup

(Nyps) was set to 21,150 YBP based on the colonisation history and divergence times estimated by Fang et al. (2018). We used the default prior and model parameters and ran 1,000,000 MCMC iterations, with a burnin of 10% and sampling every 500 steps for each dataset. Population size estimates were added to the beast log files using a custom version of the ruby script add_theta_to_log_LD.rb (https://github.com/mmatschiner/snapp_prep/blob/master/add_theta_to_log.rb). The two independent runs were assessed for stationarity and parameter convergence using Tracer version 1.6 (Rambaut et al. 2014) and ESS values exceeded 200 for all parameters. We visualised the distribution of trees using DensiTree (Figure 5g) version 2.2.5 (Bryant et al. 2012) and generated the maximum clade credibility tree using TreeAnnotator version 1.7.5 (Drummond and Rambaut 2007) using a burnin of 1000 steps. The two runs converged to three identical consensus tree topologies and therefore all trees from both runs are shown.

To model historic migration events between the same four populations we used TreeMix version 1.13 (Pickrell and Pritchard 2012). Because TreeMix is not computationally expensive to run we used the full dataset 5 (Table 3). TreeMix was run with k=10, the Icelandic marine population (Nyps) as the outgroup and between one and four migration events. The tree with the highest likelihood and least residual unexplained variance is shown (Figure 5h).

Linkage filtering

Linkage disequilibrium across the full SNP set (dataset 4) was estimated by first phasing the VCF file using the default phasing parameters in Beagle version 5.0 (Browning and Browning 2007) and then calculating pairwise R^2 estimates for all SNPs within 10,000bp of one another using the --hap-r2 flag in VCFtools. R^2 values range between 0 (no linkage) and 1 (complete linkage), and therefore a relatively conservative linkage disequilibrium threshold was set at $R^2 > 0.6$. Under this threshold, 24% of 22364 pairwise comparisons using dataset 1 identified linkage disequilibrium. Dataset 1 was then thinned using VCFtools to include only SNPs within 1000, 2000, 3000, 4000,

5000, 6000 and 7000bp of one another and phasing and R^2 calculations were recalculated for all thinned data files. SNPs filtered to at least 2000bp apart produced pairwise R^2 comparisons that identified linkage disequilibrium in <0.1% of cases and therefore thinning to >2000bp was considered acceptable to remove the majority of linked SNPs from the dataset.

Section 4c: Mitochondrial DNA analyses

To investigate the mitochondrial background of stickleback on North Uist and to identify whether the island is a meeting place for the ancient mitochondrial lineages identified by Makinen and Merila (2008), genomic DNA from the fin clips of 76 individuals (31 anadromous and 45 lagoon resident fish from five species-pair lagoons, see Table S1 for detailed sample sizes and locations) was extracted using either Qiagen DNeasy blood and tissue kits, Quanta Biosciences Extracta™ DNA prep for PCR-Tissue kits or following a proteinase K and ethanol precipitation procedure (Goldenberger et al. 1995). DNA was amplified for two mitochondrial regions; cyt *b* and a partial fragment of the D-loop CR. Amplification was carried out using the following primers: cyt *b* forward 5' ATGAAACTTTGGTTCCCTCC 3', cyt *b* reverse 5' CGCTGAGCTACTTTTGCATGT 3', CR forward 5' CCTTTAGTCCTATAATGCATG 3' and CR reverse 5' CCGTAGCCCATTAGAAAGAA 3' taken from Makinen and Merila (2008). For both regions PCR was carried out in 20µl reaction volumes consisting of 8µl nuclease-free H_20, 9µL 2X Biomix™ red reaction mix (Bioline), 1µl of both forward and reverse primers (10µM) and 1µl of template DNA (approximately 20ng). For cyt *b*, thermocycling was set up as follows: Initial denaturation at 95°C for three minutes, followed by 36 cycles of denaturation at 95°C for 30 seconds, annealing at 60°C for 30 seconds and extension at 72°C for one minute, followed by a final extension at 72°C for five minutes. For CR similar conditions were used except annealing was carried out at 53°C. Amplification success was confirmed by running samples on a 1.5% agarose gel at 110V for ~30 minutes. Cyt *b* and CR fragments were purified using ExoSAP-IT PCR product clean-up kits (Thermo-Fisher Scientific) and sequenced by Source BioScience.

Mitochondrial sequences were aligned using BioEdit version 7.2.5 (Hall 1999) and electropherograms were inspected and edited for ambiguities by eye. Cyt *b* (981bp) and CR (428bp) sequences were then concatenated using Mesquite version 3.04 (Maddison and Maddison 2015). The number of haplotypes (37) was calculated using the program DnaSP version 5.10.01 (Librado and Rozas 2009). Composite cyt *b* and CR sequences from this study (1409bp) and from a further 126 North Uist individuals studied by Rahn *et al.* (2016), see Table S1 for sampling locations and sample sizes, were subsequently trimmed and re-aligned producing a 1380bp alignment of 202 North Uist individuals. Pairwise differentiation between each pair of ecotypes were estimated by calculating the fixation index (ϕST) and associated *p*-values were estimated using 1000 permutations in the strataG version 2.0.2 (Archer et al. 2017) package in R. Probability values were corrected for multiple testing using the sequential Bonferroni method.

To determine whether the sequence divergence in North Uist stickleback corresponded to the lineages identified by Makinen and Merila (2008), the full alignment of 202 concatenated mitochondrial sequences, alongside all sequences from Makinen and Merila (2008) (downloaded from GenBank, Accession Nos. EF523391–EF525476) was analysed using a Bayesian phylogenetic approach (Figure S3) implemented in MrBayes version 3.2.2 (Ronquist and Huelsenbeck 2003), with sequence from the Broads S1 *Gasterosteus aculeatus* assembly, downloaded from Ensembl version 84.1 (Yates et al. 2016), as an outgroup. The most appropriate model of nucleotide substitution for the dataset, GTI+I+G, was determined using Akaike information criteria (AIC) (Akaike 1974) in MrModeltest version 2 (Nylander 2004), executed in PAUP* version 4.0 (Swofford 2002), and was implemented in MrBayes. Four independent runs were carried out each with eight MCMC chains 'heated' to a 'temperature' of 0.1, with a relative burnin of 50%. Analyses ran until the average standard deviation of split frequencies fell below 0.01. Traces were visually assessed and marginal likelihoods of the harmonic mean ([-3343.84] – [-3353.81]), potential scale reduction factor (PSRF) values (1.00 – 1.001 for all parameters in all runs) and effective sample sizes (ESS, >500 for all parameters in all runs) were used to confirm stationarity and parameter convergence between

independent runs in MrBayes and Tracer version 1.6 (Rambaut et al. 2014). A 50% majority rule consensus tree was then constructed from the sampled trees (Figure S3). Differences between the proportions of mitochondrial lineages across ecotypes was analysed using a chi-squared test in R with anadromous, lagoon resident and freshwater resident categories for ecotype and trans-Atlantic or European categories for mitochondrial lineage.

To further investigate genetic relationships among North Uist stickleback, a median-joining haplotype network was constructed using TCS version 1.21 (Clement et al. 2000) using 95% parsimony criteria, and alternative connections were resolved following standard methods (Crandall and Templeton 1993). Changing the treatment of gaps from 5th state to missing had no impact on the network analysis.

To date the split between the Trans-Atlantic and European mitochondrial lineages identified by the Bayesian phylogeny (Figure S3) we used coalescence-based MCMC simulations on the 76 sequences obtained in this study, implemented in IMa2 (Hey and Nielsen 2004). Nexus files were converted for IMa2 analysis using PGDSpider version 2.1.0.1 (Lischer and Excoffier 2012) . Four independent runs were carried out from different random number starting seeds. All runs assumed a HKY model of nucleotide substitution with an inheritance scalar of 0.25, as recommended for all mtDNA analyses (Hey and Nielsen 2004). Forty MCMC chains with a geometric heating scheme of 0.96 /0.9 and a burnin period of 80,000 steps were used in all runs. The upper bounds of parameter prior distributions were initially assumed based on the highest geometric mean of population mutation rates (as specified in the latest IMa2 user guide documentation), calculated using Watterson’s estimator in the DnaSP program, and were adjusted after initial runs to ensure the full probability distributions were accounted for, for all parameters. Parameter conversions to demographic units were scaled using the geometric mean of mutation rates for cyt *b* and CR, estimated using the molecular clock calibrations of Makinen and Merila (2008). For our cyt *b* sequence the mutation rate was estimated as $(2.045 \times 10^{-8}) \times 981$bp $= 2.01 \times 10^{-5}$, and for CR $(2.21 \times 10^{-8}) \times 428$bp $= 9.46 \times 10^{-6}$, giving a geometric mean of mutation rates of 1.38×10^{-5} /haplotype/year. All parameter values converged across the four

independent runs and therefore average parameter values are given (Table S5). There is, however, uncertainty surrounding any molecular clock calibrations (Warnock et al. 2012) and therefore parameter conversions to demographic units of time should be regarded as estimates only.

Table S1. Description of sample sites. Sample sizes are shown for morphological (N_{morph}), mitochondrial (N_{mt}), RAD-seq (N_{RAD}) and *Eda* (N_{Eda}) analyses. Sample sizes are given for lagoon resident fish, anadromous fish (curved parentheses) and freshwater resident fish (square parentheses). Sampling years in which stickleback were taken for morphological and/or genetic analysis ([s]) and in which sediment core samples were collected ([c], bold text) are shown. Stickleback samples collected in 2010 and 2011 were obtained by Rahn et al. (2016). Samples for RAD analysis were collected by Magalhaes et al. (2016). Sampling locations are given by latitude followed by longitude. Sal refers to salinity classifications, Fr: freshwater, Ma: marine, Br: brackish.

Lake ID	N morph	N mt	N RAD	N Eda	Year	Sal	Location
Acha		[5]			2010[s]	Fr	57°35′45"N; 7°23′42"W
Ardh		5 (6)			2011[s]	Fr	57°34′48"N; 7°24′48"W
Bhar		[5]			2011[s]	Fr	57°34′24"N; 7°17′42"W
Bhoi					**2015[c]**	Ma	57°38'37"N; 7°12'6"W
Buai		[5]			2010[s]	Fr	57°38'49"N; 7°11'51"W
Clac		5 (5)			2011[s]	Fr	57°38′14"N; 7°24′45"W
Crei					**2013[c]**	Fr	57°38'41"N; 7°13'33"W
Daim		[4]			2011[s]	Fr	57°35′35"N; 7°12′35"W
Dheo	20 (14)			(2)	**2015[c]**	Ma	57°38'26"N; 7° 9'58"W
Dubh		[5]			2011[s]	Fr	57°34′54"N; 7°24′12"W
Duin	20 (19)	10 (3)		6 (3)	2015[s]	Br	57°38'35"N; 7°12'40"W
Eile		[5]			2011[s]	Fr	57°34′24"N; 7°15′30"W
Eubh		[5]			2011[s]	Fr	57°37′6"N; 7°29′42"W
Faik[1]	19 (19)	15 (17)		13	2011[s], **2015[c,s]**	Br	57°38'7"N; 7°12'54"W
Geir		[5]			2011[s]	Fr	57°38′34"N; 7°25′18"W
Grog		[5]			2011[s]	Fr	57°36′54"N; 7°30′40"W
Host		[5]			2011[s]	Fr	57°37′40"N; 7°29′18"W
Iala					**2013[c]**	Fr	57°37'54"N: 7°12'31"W
Maga		[5]			2010[s]	Fr	57°36′10"N; 7°28′54"W
Maig		[5]			2011[s]	Fr	57°35′42"N; 7°12′6"W
Mgbh		[5]			2010[s]	Fr	57°36′6"N; 7°24′36"W
Moin		[5]			2011[s]	Fr	57°35′42"N; 7°25′48"W
Mora		[5]			2011[s]	Fr	57°34′30"N; 7°16′18"W
Nyps			(19)		2013[s]	Ma	65°46'19"N; 14°50'9"W
Obse	19 (20)	10 (10)	18 (16)	10 (10)	**2013[c]**, **2015[c,s]**	Br	57°36'6"N; 7°10'22"W
Olav		[5]			2011[s]	Fr	57°39′8"N; 7°26′48"W
Port					**2015[c]**	Ma	57°38'1"N; 7° 7'10"W
Sann		[5]			2010[s], 2011[s]	Fr	57°35′12"N; 7°27′48"W
Scad		[4]	[17]		2011[s], 2013[s]	Fr	57°35′6"N; 7°14′10"W
Strm	44 (4)	5 (2)			**2015[c,s]**	Br	57°36'36"N; 7°11'1"W
Torm		[5]			2010[s]	Fr	57°33′45"N; 7°19′1"W
Trum	20 (20)	10 (6)		5 (6)	2015[s]	Br	57°39'9"N; 7°14'35"W

[1] referred to as “Aileodair” by Rahn et al. (2016)

Table S2. Diatom taxa identified in Obse, North Uist. Salinity classifications follow Van Dam et al. (1994).

Diatom taxa	Total number of valves identified	Salinity classification
Acnanthes bahusiensis	3	freshwater
Acnanthes curtissima	31	freshwater
Acnanthes flexella	70	freshwater
Acnanthes helvetica	131	freshwater
Acnanthes impexiformis	2	freshwater
Acnanthes laevis	89	freshwater
Acnanthes minutissima	708	freshwater
Acnanthes oblongella	8	freshwater
Acnanthes pseudoswazi	4	freshwater
Acnanthes pusilla	8	freshwater
Acnanthes rosii	4	freshwater
Acnanthes subatomoides	4	freshwater
Amphora coffeaeformis	10	freshwater
Amphora libyaca	4	freshwater
Amphora vetenta	44	freshwater
Amphipleura pellucida	2	freshwater
Anomoeoneis brachysira	390	freshwater
Anomoeoneis brachysira var. Zellenis	18	freshwater
Anomoeoneis sphaerophora	4	freshwater
Anomoeoneis styriaca	114	freshwater
Anomoeoneis vitrea	1029	freshwater
Aulacoseira alpigena	1	freshwater
Aulacoseira lacustris	1	freshwater
Aulacoseira lirata	96	freshwater
Caloneis lauta	1	freshwater
Caloneis silicula	3	freshwater
Cocconeis disculus	5	freshwater
Cocconeis neodiminuta	36	saltwater
Cocconeis placentula	11	freshwater
Cocconeis placentula var. pseudolineata	2	freshwater
Cocconeis placentula var. lineata	1	freshwater
Cocconeis scutellum	25	saltwater
Cyclotella antiqua	28	freshwater
Cyclotella atomus	469	freshwater
Cyclotella bodanica	1	freshwater
Cymbella ampicephala	3	freshwater
Cymbella angustata	46	freshwater
Cymbella caespitosa	1	freshwater
Cymbella cesatii	189	freshwater
Cymbella cistula	2	freshwater
Cymbella cuspidata	1	freshwater

Diatom taxa	Total number of valves identified	Salinity classification
Cymbella cymbiformis	30	freshwater
Cymbella descripta	7	freshwater
Cymbella eligensis	1	freshwater
Cymbella falaisensis	82	freshwater
Cymbella gaeumannii	32	freshwater
Cymbella gracilis	288	freshwater
Cymbella incerta	1	freshwater
Cymbella mesiana	1	freshwater
Cymbella microcephala	90	freshwater
Cymbella minuta	65	freshwater
Cymbella naviculacea	30	freshwater
Cymbella paucistriata	1	freshwater
Cymbella perpusilla	6	freshwater
Cymbella pseudoaffins	2	freshwater
Cymbella silesiaca	66	freshwater
Cymbella subcuspidata	8	freshwater
Cymbella symonsenii	3	freshwater
Diatoma mesodon	7	freshwater
Diatoma tenuis	1	freshwater
Diploneis finnica	3	freshwater
Diploneis interrupta	2	freshwater
Diploneis modica	1	freshwater
Diploneis pseudovalis	1	freshwater
Epithemia sorex	126	freshwater
Eunotia arcus	176	freshwater
Eunotia bilunaris	27	freshwater
Eunotia bilunaris var. bilunaris	3	freshwater
Eunotia bilunaris var. mucophila	2	freshwater
Eunotia circumborealis	1	freshwater
Eunotia exigua	3	freshwater
Eunotia glacialis	1	freshwater
Eunotia implicata	141	freshwater
Eunotia incisa	16	freshwater
Eunotia meisteri	1	freshwater
Eunotia monodon	2	freshwater
Eunotia pectinalis var. undulata	3	freshwater
Eunotia praerupta	1	freshwater
Eunotia septentrionalis	10	freshwater
Fragiliaria alpestris	1	freshwater
Fragilaria biceps	3	freshwater
Fragilaria brevistriata	3	freshwater
Fragilaria capucina	10.5	freshwater
Fragilaria capucina var. austriaca	7	freshwater
Fragilaria construens	6	freshwater

Diatom taxa	Total number of valves identified	Salinity classification
Fragilaria cyclopum	1	freshwater
Fragilaria exigua	2202	freshwater
Fragilaria parasitica var. subconstricta	12	freshwater
Frustulia rhomboides	785	freshwater
Gomphonema acuminatum	50	freshwater
Gomphonema angustatum	6	freshwater
Gomphonema hebridense	8	freshwater
Gomphonema parvulum	28	freshwater
Gomphonema subtile	2	freshwater
Gyrosigma acuminatum	0.5	freshwater
Navicula amphibola	1	freshwater
Navicula angusta	7	freshwater
Navicula capitatoradiata	6	freshwater
Navicula cocconeiformis	33	freshwater
Navicula concentrica	2	freshwater
Navicula cryptocephala	188	freshwater
Navicula cryptonella	4	freshwater
Navicula cuspidata	1	freshwater
Navicula detenta	15	freshwater
Navicula globulifera	14	freshwater
Navicula halophila	208	freshwater
Navicula jaernfefeltii	1	freshwater
Navicula leptostriata	31	freshwater
Navicula mediocris	10	freshwater
Navicula menisculus	1	freshwater
Navicula minima	2	freshwater
Navicula miniscula	2	freshwater
Navicula oblonga	1	freshwater
Navicula placentula	2	freshwater
Navicula platystoma	1	freshwater
Navicula protracta	4	freshwater
Navicula pupla	25	freshwater
Navicula pupla var. aquaeductae	2	freshwater
Navicula radiosa	280	freshwater
Navicula rhynchocephala	33	freshwater
Navicula subplacentula	3	freshwater
Navicula subtilissima	81	freshwater
Navicula variostriata	3	freshwater
Navicula veneta	3	freshwater
Navicula viridula var. linearis	1	freshwater
Neidium ampliatum	4	freshwater
Neidium bergii	8	freshwater
Neidium dubium	3	freshwater
Nitzschia frustulum	113	freshwater

Diatom taxa	Total number of valves identified	Salinity classification
Nitzschia levidensis var. victoriae	1	freshwater
Nitzschia nana	103	freshwater
Nitzschia paleaca	56	freshwater
Nitzschia perminuta	16	freshwater
Nitzschia rosenstockii	2	freshwater
Pinnularia braunii	1	freshwater
Pinnularia gibba	23	freshwater
Pinnularia intermedia	1	freshwater
Pinnularia interupta	72	freshwater
Pinnularia legumen	1	freshwater
Pinnularia maior	2	freshwater
Pinnularia microstauron	13	freshwater
Pinnularia microstauron var. brebissonii	3	freshwater
Pinnularia subcapita	7	freshwater
Pinnularia virdis	14	freshwater
Rhopalodia gibba	25	freshwater
Rhopalodia rupestris	101	freshwater
Stauroneis phoicenteron	30	freshwater
Surirella amphioxys	1	freshwater
Surirella splendida	2	freshwater
Taballaria floculosa	158	freshwater
Unknown	3	freshwater

346

Table S3. Radiocarbon dates. Radiocarbon and calibrated radiocarbon dates for six macrofossil samples from the Faik long core. Depth corresponds to absolute depth from the surface of the lake bed, Rc: radiocarbon.

Lagoon	Drive	Depth (cm)	Rc age (years BP ± 1σ)	Calibrated Rc age (years BP ± 1σ)	Macrofossil material	Publication code
Faik	D3	74-75	2793 ± 35	2900 ± 41	plant	SUERC-67962
Faik	D3	92-93	3209 ± 35	3430 ± 30	plant	SUERC-67963
Faik	D3	112-113	3329 ± 35	3560 ± 65	plant	SUERC-67967
Faik	D3	152-153	5815 ± 40	6168 ± 51	plant	UCIAMS-176364
Faik	D5	303-305	10039 ± 41	11562 ± 152	plant	SUERC-67968
Faik	D5	319-320	11294 ± 44	13193 ± 96	plant	SUERC-67969

Table S4

Outlier loci likely under divergent selection between anadromous and lagoon resident stickleback populations. Table shows loci detected by POPULATIONS (P<0.99) and BayeScan (P<0.99), the genes in which outlier SNPs are located and their associated proteins.

Lg	SNP position	POP	BS	Ensembl gene ID	Protein
I	5701593	Y		ENSGACG00000007444	Delta/notch-like EGF repeat containing (Dner)
I	21595692	Y	Y	ENSGACG00000014280	Insulin-like growth factor binding protein 2a (Igfbp2a)
I	21607623	Y	Y	ENSGACG00000018903	Serine/threonine kinase 11 interacting protein (Stk11ip)
I	21629854	Y	Y	No hit	No hit
I	21770493	Y	Y	ENSGACT00000018966	Unknown
I	21798529	Y	Y	No hit	No hit
I	21798582	Y		No hit	No hit
II	7956000	Y		ENSGACG00000015374	Unknown
II	11647533	Y	Y	No hit	No hit
II	12015572	Y		ENSGACG00000015955	methenyltetrahydrofolate synthetase domain containing (Mthfsd)
II	13058876	Y		ENSGACG00000016050	CUB and Sushi multiple domains 1 (CSMD1)
II	13068161	Y	Y	ENSGACG00000016050	CUB and Sushi multiple domains 1 (CSMD1)
II	14483200	Y		No hit	No hit
II	14506528	Y		ENSGACG00000016281	growth regulating estrogen receptor binding 1 (Greb1)
III	9127800	Y		ENSGACG00000015994	solute carrier family 35 (UDP-N-acetylglucosamine (UDP-GlcNAc) transporter), member A3b (Slc35a3b)
III	9599272	Y		ENSGACG00000016156	SH3-domain GRB2-like (endophilin) interacting protein 1b
IV	4930687	Y	Y	ENSGACG00000016877	Diacylglcerol kinase theta (DGKQ)
IV	8643876	Y	Y	No hit	No hit
IV	12198507	Y	Y	No hit	No hit
IV	12808630	Y	Y	ENSGACG00000018311, ENSGACG00000018298	Ectodysplasin A (Eda), Vacuolar H+-ATPase homolog (S. cerevisiae (Vma21)
IV	12808717	Y	Y	ENSGACG00000018311, ENSGACG00000018298	Ectodysplasin A (Eda), Vacuolar H+-ATPase homolog (S. cerevisiae (Vma21)

Lg	SNP position	POP	BS	Ensembl gene ID	Protein
IV	12812539	Y		ENSGACG00000018312, ENSGACG00000018298	Unknown, Vacuolar H+-ATPase homolog (S. cerevisiae (Vma21)
IV	12829883	Y	Y	ENSGACG00000018298	Vacuolar H+-ATPase homolog (S. cerevisiae (Vma21)
IV	12831052	Y	Y	ENSGACG00000018298	Vacuolar H+-ATPase homolog (S. cerevisiae (Vma21)
IV	12831136	Y	Y	ENSGACG00000018298	Vacuolar H+-ATPase homolog (S. cerevisiae (Vma21)
IV	20443675	Y		No hit	No hit
VI	375067	Y		ENSGACG00000002297	Cadherin 30 (Cdh30)
VI	8369447	Y		No hit	No hit
VII	15030323	Y		No hit	No hit
VIII	3022825	Y		No hit	No hit
VIII	8270922	Y	Y	ENSGACG00000007270	Unknown
VIII	8270959	Y	Y	ENSGACG00000007270	Unknown
VIII	8859227	Y		ENSGACG00000007843	protein tyrosine phosphatase, receptor type, C (Ptprc)
VIII	11219937	Y		No hit	No hit
X	5510289	Y		No hit	No hit
X	5510380	Y		No hit	No hit
XI	8556295	Y		ENSGACG00000010824	Sidekick cell adhesion molecule 2 (SDK2)
XI	8572077	Y	Y	ENSGACG00000010824	Sidekick cell adhesion molecule 2 (SDK2)
XI	8988005	Y	Y	ENSGACG00000010994	BAH domain and coiled-coil containing 1a (Bahcc1a)
XIII	6797023	Y		No hit	No hit
XIII	10696924	Y		ENSGACG00000010369	si:dkey-245n4.2
XIII	12601544	Y		No hit	No hit
XVII	2506925	Y		ENSGACG00000004607	eukaryotic translation initiation factor 4 gamma, 3a (eif4g3a)
XVII	6890281	Y		ENSGACG00000007882	ubiquitin-conjugating enzyme E2, J2 (UBC6 homolog, yeast)
XX	6030073	Y		ENSGACG00000006371	5-azacytidine induced 2 (Azi2)
XX	9124797	Y		ENSGACG00000007711	nephrosis 1, congenital, Finnish type (nephrin) (Nphs1)
XX	9270864	Y		ENSGACG00000007921	Unknown
XX	10210859	Y		No hit	No hit
XX	12102108	Y		ENSGACG00000010601	recoverin 2 (Rcvrn2)
XXI	1425494	Y		No hit	No hit
S_27	3836305	Y		ENSGACG00000001327	prostaglandin I2 (prostacyclin) synthase (Ptgis)

Abbreviations: Lg, linkage group; POP, POPULATIONS; BS, BayeScan; S, scaffold.

Table S5

Posterior probability of parameter estimates of the IMa2 coalescence simulations and their corresponding parameter conversions. Table shows parameter values (averaged across four independent runs) for divergence times (*t*) and time since the most recent common ancestor (TMRCA) and their conversion to years, which were calculated based on a generation time of 1 year and a geometric mean of substitution rates for cyt b and CR combined of 1.38 x 10^{-5} gene/year. HPD: higher posterior density intervals. High points and smoothed high points for all posterior probabilities were identical and thus only high points are shown.

**Trans-Atlantic vs European**	***t***	***t* (years)**	**TMRCA**	**TMRCA (years)**
High point	1.643	119,114	2.188	158,515
Lower 95% HPD	0.659	47,773	1.125	81,522
Upper 95% HPD	4.161	301,773	4.150	300,725

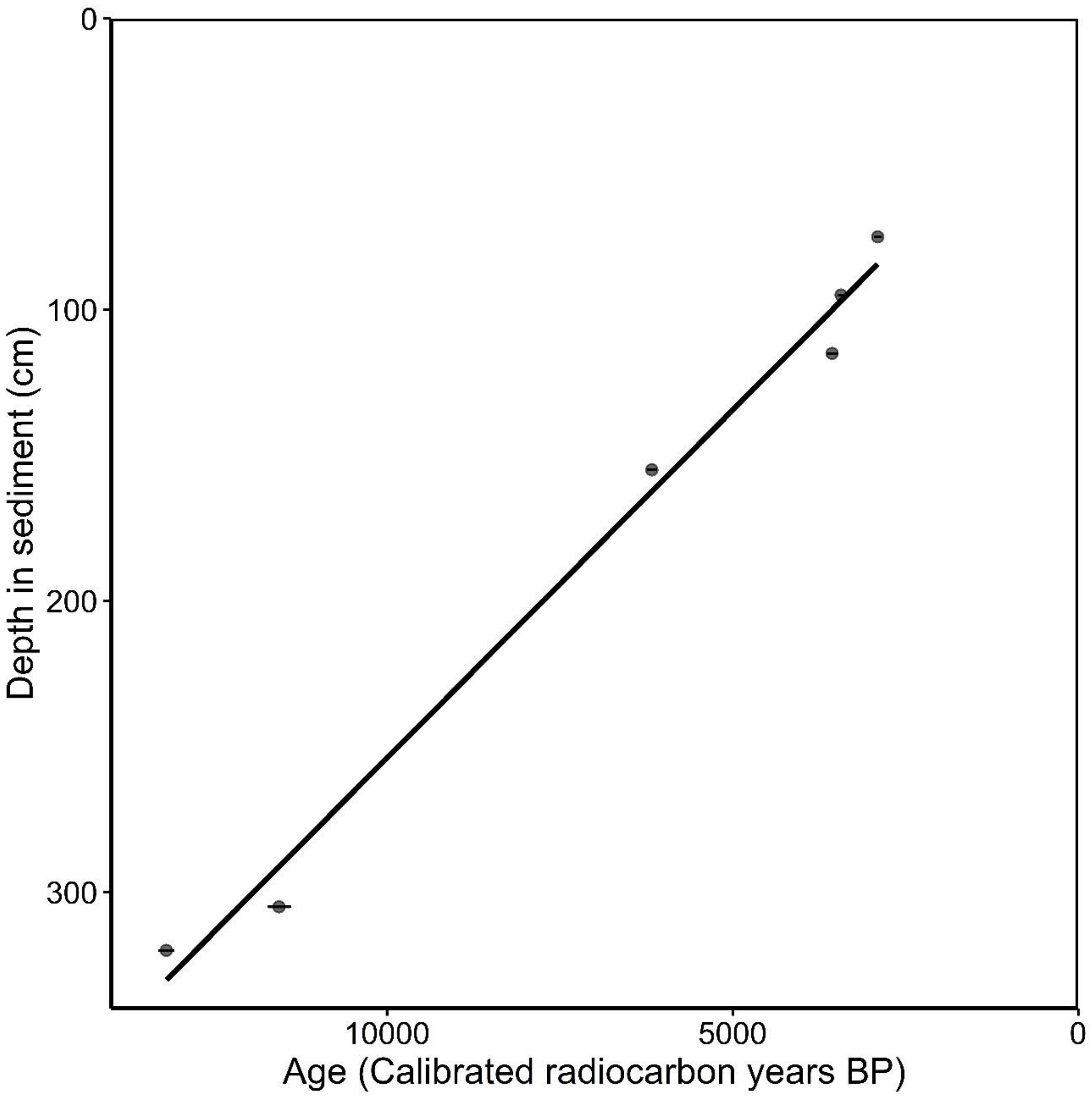

Figure S1. Depth-age correlations for sediment deposits in Faik. Calibrated radiocarbon dates (grey circles) with the standard deviation indicated by associated error bars. The lm fit is indicated by the solid black line and represents the mean sedimentation rate used to estimate the age of sediment deposits in other long cores.

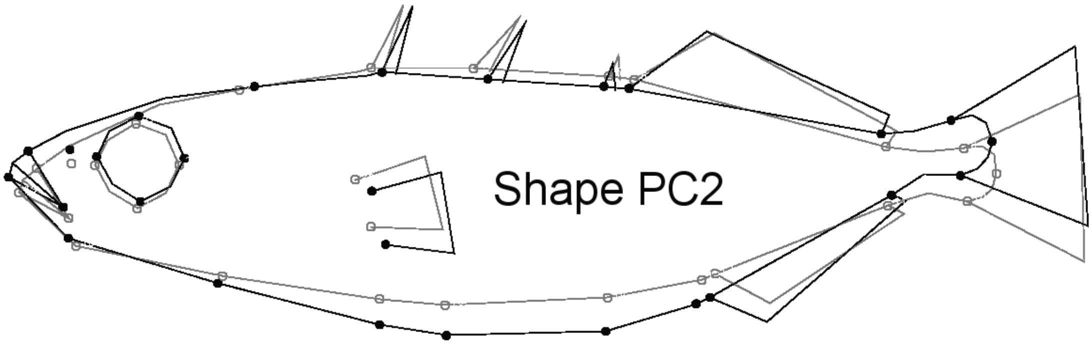

Figure S2. Shape PC2 warped outline drawing. Warped outline drawing shows that shape PC2 largely described specimen bending and therefore was not considered further in our analyses.

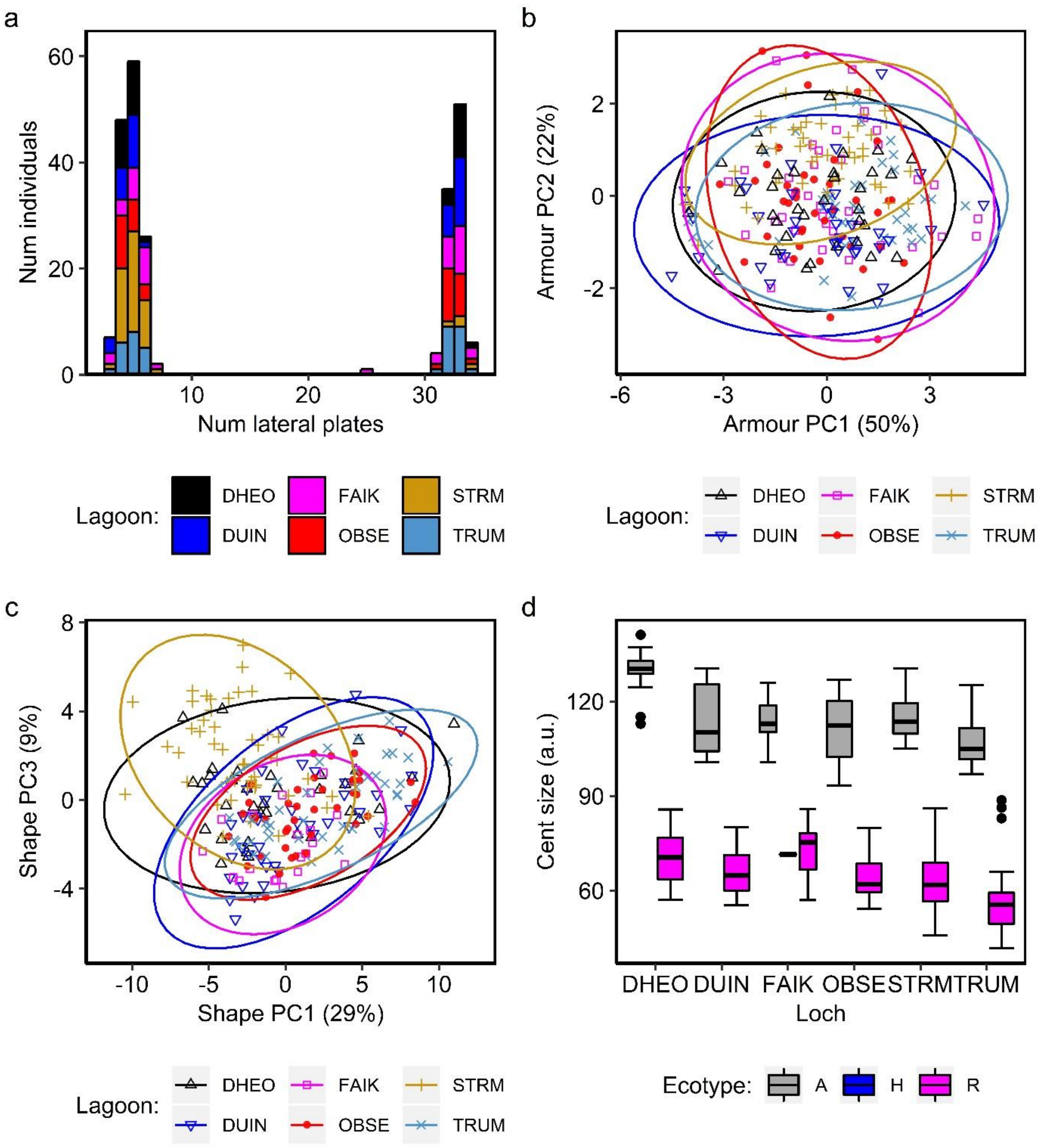

Figure S3. Lake specific morphological variation in North Uist species-pairs. (a) histogram showing lateral plate counts. (b) Distribution of phenotypes and their associated 95% confidence ellipses in a principal components analysis (PCA) of all size standardised, continuous body armour variables. Principal component 1 (Armour PC1, explaining 50% of variation in the data) described an increase in the size of all armour variables and Armour PC2 (explaining 22% of variation in the data) described a relative increase in the size of the pelvis and decrease in spine length. (c) Distribution of phenotypes and their associated 95% confidence ellipses in a PCA of 56 body shape variables. Shape PC1 (explaining 29% of variation in the data) described an increase in posterior body depth, mouth size, and a more rearward positioning of the pectoral fin. Shape PC3 (explaining 9% of variation in the data) largely described an increase in anterior body depth and shortening of the caudal peduncle. (d) Box-plots showing centroid size, with error bars representing the standard error of the mean (*SEM*). (a) – (d) are based on analyses of 239 individuals from 6 lakes containing species-pairs (Figure 1d, Table S1).

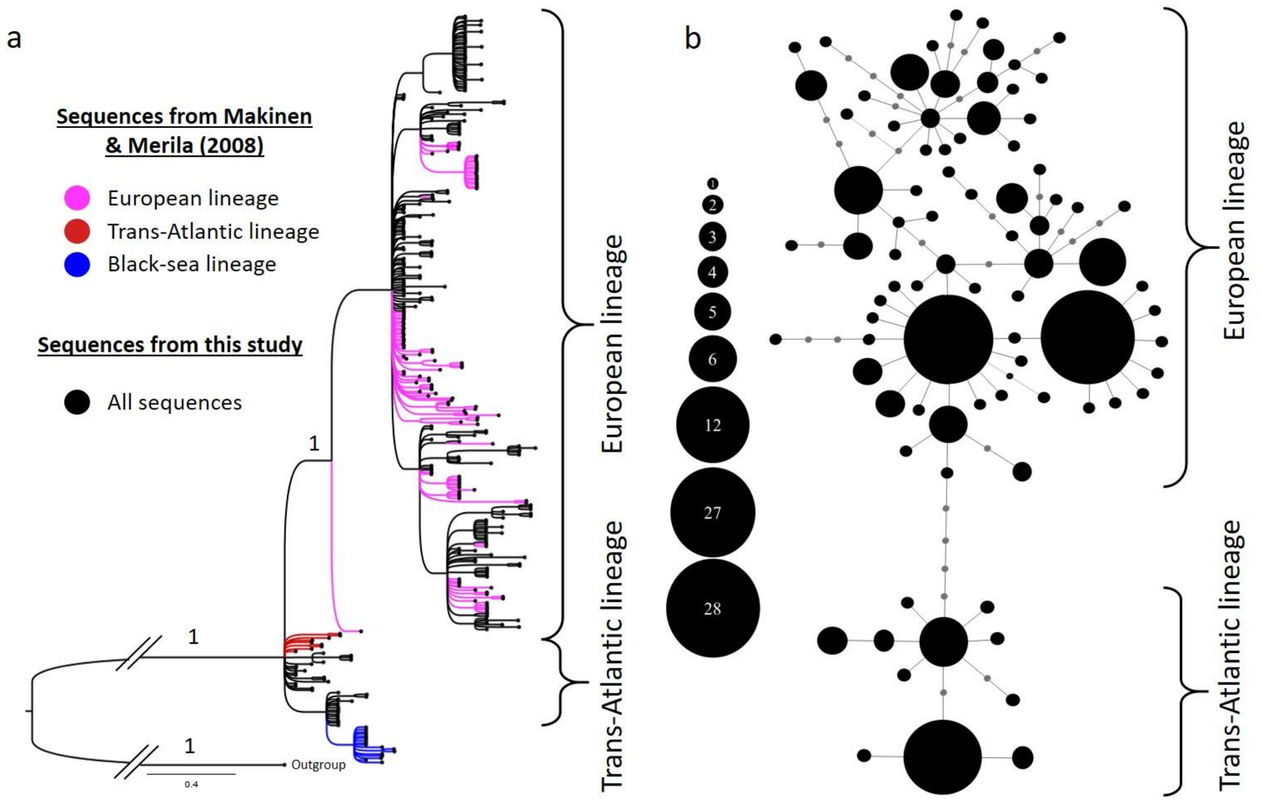

Figure S4. Mt genetic analyses. (a) Bayesian 50% majority rule consensus phylogeny of 76 North Uist stickleback sequenced in this study, 126 North Uist sequences from Rahn et al. (2016) and 176 individuals sequenced by Makinen and Merila (2008), based on concatenated cytochrome *b* (cyt *b*) and mitochondrial control region (CR) sequences. Mitochondrial sequences from the Broads S1 *Gasterosteus aculeatus* assembly (Ensembl) were used as an outgroup. All posterior probabilities > 0.98 are given on branches. (b) Median-joining haplotype network of 76 North Uist stickleback sequenced in this study and 126 North Uist sequences from Rahn et al. (2016), constructed using TCS. Black circles indicate haplotypes, grey circles indicate single mutational steps. Major mitochondrial lineages first identified by Makinen and Merila (2008) are indicated with parentheses in (a) and (b).

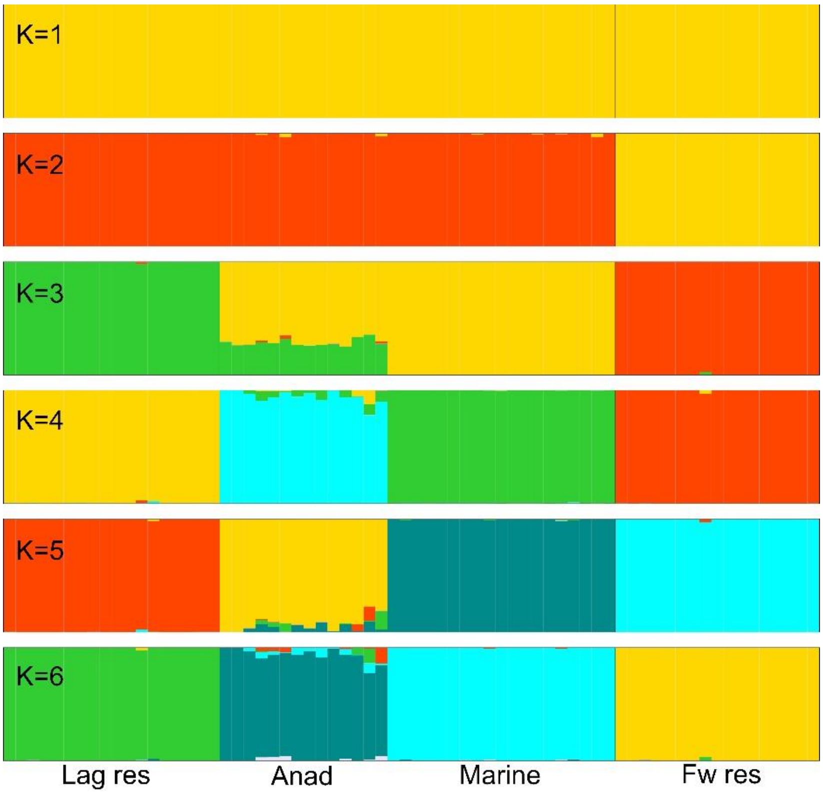

Figure S5. Structure plots. Structure plots showing the estimated population structures with between one and six clusters specified (K=1 to K=6). For each value of K the structure plot with the highest likelihood of five independent runs is shown. Lag res: lagoon resident, Anad: anadromous, Fw res: freshwater resident.

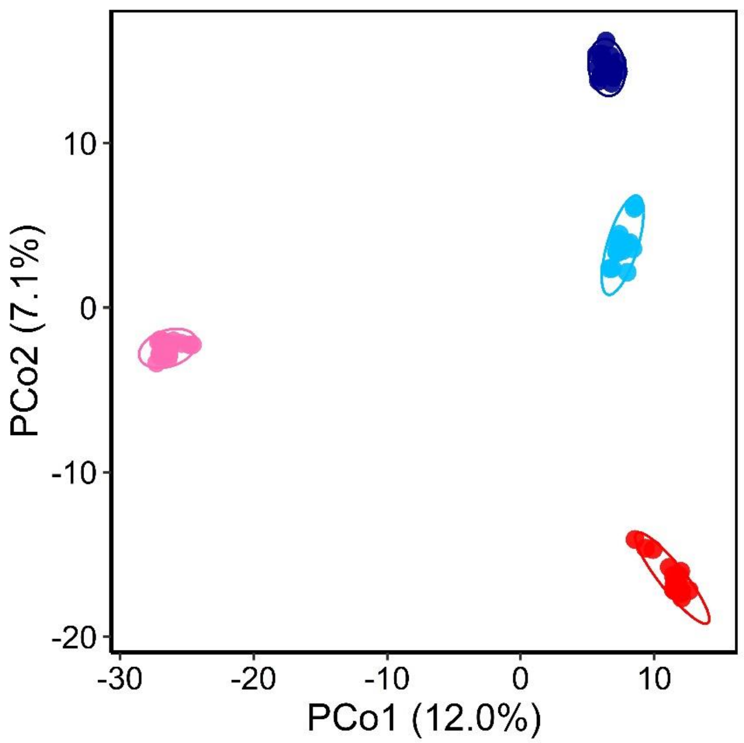

Figure S6 Principal coordinate analysis (PCoA) on neutral SNPs. Plot showing the first two principal coordinate axes (and the proportion of total genetic variation explained by each axis) of a PCoA on SNP dataset 4b (which excluded all SNPs identified as putatively under selection between anadromous and lagoon resident fish in our outlier analyses, Figure 4b & Figure 4c, leaving only putatively neutral SNPs).

References

Akaike H. 1974. A new look at the statistical model identification. *IEEE Transactions on automatic control* 19:716-723.

Archer FI, Adams PE, Schneiders BB. 2017. STRATAG: An R package for manipulating, summarizing and analysing population genetic data. *Molecular Ecology Resources* 17:5-11.

Battarbee RW, Jones VJ, Flower RJ, Cameron NG, Bennion H, Carvalho L, Juggins S. 2001. Diatoms. In: Smol JP, Birks HJB, Last WM, Bradley RS, Alverson K, editors. Tracking environmental change using lake sediments. Netherlands: Springer. p. 155-202.

Blaauw M, Andres Christen J. 2011. Flexible Paleoclimate Age-Depth Models Using an Autoregressive Gamma Process. *Bayesian Analysis* 6:457-474.

Blackmon H, Chromartie, Anya. 2015. evobir: evobiRv1.1. *Zenodo*.

Browning SR, Browning BL. 2007. Rapid and accurate haplotype phasing and missing-data inference for whole-genome association studies by use of localized haplotype clustering. *Am. J. Hum. Genet.* 81:1084-1097.

Bryant D, Bouckaert R, Felsenstein J, Rosenberg NA, RoyChoudhury A. 2012. Inferring Species Trees Directly from Biallelic Genetic Markers: Bypassing Gene Trees in a Full Coalescent Analysis. *Molecular Biology and Evolution* 29:1917-1932.

Catchen J, Hohenlohe PA, Bassham S, Amores A, Cresko WA. 2013. Stacks: an analysis tool set for population genomics. *Molecular Ecology* 22:3124-3140.

Chague-Goff C, Chan JCH, Goff J, Gadd P. 2016. Late Holocene record of environmental changes, cyclones and tsunamis in a coastal lake, Mangaia, Cook Islands. *Isl. Arc.* 25:333-349.

Clement M, Posada D, Crandall KA. 2000. TCS: a computer program to estimate gene genealogies. *Molecular Ecology* 9:1657-1659.

Colosimo PF, Hosemann KE, Balabhadra S, Villarreal G, Dickson M, Grimwood J, Schmutz J, Myers RM, Schluter D, Kingsley DM. 2005. Widespread parallel evolution in sticklebacks by repeated fixation of ectodysplasin alleles. *Science* 307:1928-1933.

Crandall KA, Templeton AR. 1993. Empirical tests of some predictions from coalescent theory with applications to intraspecific phylogeny reconstruction. *Genetics* 134:959-969.

Danecek P, Auton A, Abecasis G, Albers CA, Banks E, DePristo MA, Handsaker RE, Lunter G, Marth GT, Sherry ST et al. 2011. The variant call format and VCFtools. *Bioinformatics* 27:2156-2158.

Drummond AJ, Rambaut A. 2007. BEAST: Bayesian evolutionary analysis by sampling trees. *Bmc Evolutionary Biology* 7:8.

Durand EY, Patterson N, Reich D, Slatkin M. 2011. Testing for Ancient Admixture between Closely Related Populations. *Molecular Biology and Evolution* 28:2239-2252.

Falush D, Stephens M, Pritchard JK. 2003. Inference of population structure using multilocus genotype data: Linked loci and correlated allele frequencies. *Genetics* 164:1567-1587.

Fang BH, Merila J, Ribeiro F, Alexandre CM, Momigliano P. 2018. Worldwide phylogeny of three-spined sticklebacks. *Molecular Phylogenetics and Evolution* 127:613-625.

Filikci B, Eris KK, Cagatay N, Sabuncu A, Polonia A. 2017. Late glacial to Holocene water level and climate changes in the Gulf of Gemlik, Sea of Marmara: evidence from multi-proxy data. *Geo-Mar. Lett.* 37:501-513.

Foll M, Gaggiotti O. 2008. A Genome-Scan Method to Identify Selected Loci Appropriate for Both Dominant and Codominant Markers: A Bayesian Perspective. *Genetics* 180:977-993.

Goldenberger D, Perschil I, Ritzler M, Altwegg M. 1995. A simple universal DNA extraction procedure using sds and proteinase-k is compatible with direct pcr amplification. *Pcr-Methods and Applications* 4:368-370.

Haenel Q, Roesti M, Moser D, Maccoll ADC, Berner D. 2019. Predictable genome-wide sorting of standing genetic variation during parallel adaptation to basic versus acidic environments in stickleback fish. *Evolution Letters* 0:1-15.

Hall TA. 1999. BioEdit: a user-friendly biological sequence alignment editor and analysis program for Windows 95/98/NT. *Nucleic Acid Symposium Series* 41:95-98.

Hey J, Nielsen R. 2004. Multilocus methods for estimating population sizes, migration rates and divergence time, with applications to the divergence of *Drosophila pseudoobscura* and *D-persimilis*. *Genetics* 167:747-760.

Jakobsson M, Rosenberg NA. 2007. CLUMPP: a cluster matching and permutation program for dealing with label switching and multimodality in analysis of population structure. *Bioinformatics* 23:1801-1806.

Jombart T. 2008. adegenet: a R package for the multivariate analysis of genetic markers. *Bioinformatics* 24:1403-1405.

Klingenberg CP. 2011. MorphoJ: an integrated software package for geometric morphometrics. *Molecular Ecology Resources* 11:353-357.

Krammer K, Lange-Bertalot H. 1988a. Bacillariophyceae 1. Teil Naviculaceae. Heidelberg, Berlin: Spektrum Akademischer Verlag.

Krammer K, Lange-Bertalot H. 1988b. Bacillariophyceae 2. Teil Bacillariaceae, Epithemiaceae, Surirellaceae. Heidelberg, Berlin: Spektrum Akademischer Verlag.

Krammer K, Lange-Bertalot H. 1988c. Bacillariophyceae 3. Teil Centrales, Fragilariaceae, Eunotiaceae. Heidelberg, Berlin: Spektrum Akademischer Verlag.

Lawson DJ, Hellenthal G, Myers S, Falush D. 2012. Inference of Population Structure using Dense Haplotype Data. *Plos Genetics* 8.

Li H, Handsaker B, Wysoker A, Fennell T, Ruan J, Homer N, Marth G, Abecasis G, Durbin R, Genome Project Data P. 2009. The Sequence Alignment/Map format and SAMtools. *Bioinformatics* 25:2078-2079.

Librado P, Rozas J. 2009. DnaSP v5: a software for comprehensive analysis of DNA polymorphism data. *Bioinformatics* 25:1451-1452.

Lischer HEL, Excoffier L. 2012. PGDSpider: an automated data conversion tool for connecting population genetics and genomics programs. *Bioinformatics* 28:298-299.

Mesquite: a modular system for evolutionary analysis. Version 3.04 [Internet]. 2015. Available from: http://mesquiteproject.org

Magalhaes IS, Agostino DD, Hohenlohe PA, Maccoll ADC. 2016. The ecology of an adaptive radiation of three-spined stickleback from North Uist, Scotland. *Molecular Ecology* 25:4319-4336.

Makinen HS, Merila J. 2008. Mitochondrial DNA phylogeography of the three-spined stickleback (*Gasterosteus aculeatus*) in Europe - Evidence for multiple glacial refugia. *Molecular Phylogenetics and Evolution* 46:167-182.

Malinsky M, Trucchi E, Lawson DJ, Falush D. 2018. RADpainter and fineRADstructure Population Inference from RADseq Data. *Molecular Biology and Evolution* 35:1284-1290.

McFadden D. 1973. Conditional logit analysis of qualitative choice behavior. In: Zarembka P, editor. Frontiers in Econometrics. New York: Academic Press. p. 105-142.

Nylander JAA. 2004. MrModeltest V2. In. Evolutionary Biology Centre, Uppsala University.: Program is distributed by the author.

Peichel CL, Nereng KS, Ohgi KA, Cole BLE, Colosimo PF, Buerkle CA, Schluter D, Kingsley DM. 2001. The genetic architecture of divergence between threespine stickleback species. *Nature* 414:901-905.

Pickrell JK, Pritchard JK. 2012. Inference of Population Splits and Mixtures from Genome-Wide Allele Frequency Data. *Plos Genetics* 8:17.

Pritchard JK, Stephens M, Donnelly P. 2000. Inference of population structure using multilocus genotype data. *Genetics* 155:945-959.

R.Core.Team. 2017. R: a language and environment for statistical computing. Version Version 3.4.1. Vienna, Austria: R Foundation for Statistical Computing.

Rahn AK, Krassmann J, Tsobanidis K, MacColl ADC, Bakker TCM. 2016. Strong neutral genetic differentiation in a host, but not in its parasite. *Infection Genetics and Evolution* 44:261-271.

Rambaut A, Suchard MA, Xi D, Drummond AJ. 2014. Tracer v1.6. Available from http://beast.bio.ed.ac.uk/Tracer.

Reist JD. 1986. An empirical-evaluation of coefficients used in residual and allometric adjustment of size covariation. *Canadian Journal of Zoology-Revue Canadienne De Zoologie* 64:1363-1368.

Rohlf FJ. 2010. TpsDig, version 2.16. *Department of Ecology and Evolution, State University of New York at Stony Brook*:Available at http://life.bio.sunysb.edu/morph/.

Ronquist F, Huelsenbeck JP. 2003. MRBAYES 3: Bayesian phylogenetic inference under mixed models *Bioinformatics* 19:1572-1574.

Schneider CA, Rasband WS, Eliceiri KW. 2012. NIH Image to ImageJ: 25 years of image analysis. *Nature Methods* 9:671-675.

Snoeijs P. 1993. Intercalibration and distribution of diatom species in the Baltic Sea. Sweden: Opulus Press Uppsala.

Swofford DL. 2002. PAUP*. Phylogenetics Analysis Using Parsimony (*and Other Methods). Version 4. Sunderland, Massachusetts: Sinauer Associates.

Van Dam H, Mertens A, Sinkeldam J. 1994. A coded checklist and ecological indicator values of freshwater diatoms from the Netherlands. *Netherlands Journal of Aquatic Ecology* 28:117-133.

Venables WN, Ripley BD. 2002. Modern applied statistics with S. New York: Springer.

Walker JA, Bell MA. 2000. Net evolutionary trajectories of body shape evolution within a microgeographic radiation of threespine sticklebacks (*Gasterosteus aculeatus*). *Journal of Zoology* 252:293-302.

Warnock RCM, Yang Z, Donoghue PCJ. 2012. Exploring uncertainty in the calibration of the molecular clock. *Biology Letters* 8:156-159.

Weninger B, Joris O, Danzeglcke U. 2007. CalPal: cologne radiocarbon calibration & palaeoclimate research package. http://ww.calpal.de/.

Yang J, Benyamin B, McEvoy BP, Gordon S, Henders AK, Nyholt DR, Madden PA, Heath AC, Martin NG, Montgomery GW et al. 2010. Common SNPs explain a large proportion of the heritability for human height. *Nature Genetics* 42:565-U131.

Yates A, Akanni W, Amode MR, Barrell D, Billis K, Carvalho-Silva D, Cummins C, Clapham P, Fitzgerald S, Gil L et al. 2016. Ensembl 2016. *Nucleic Acids Research* 44:710-716.

Ziegler M, Jilbert T, de Lange GJ, Lourens LJ, Reichart GJ. 2008. Bromine counts from XRF scanning as an estimate of the marine organic carbon content of sediment cores. *Geochem. Geophys. Geosyst.* 9.
